# Effects of even- versus uneven-aged tree species (*Pinus massoniana* and *Castanopsis hystrix*) mixing on microbial communities across bulk soil, rhizosphere soil, and fine roots

**DOI:** 10.3389/fmicb.2025.1640866

**Published:** 2025-12-03

**Authors:** Runhong Liu, Yuxin Bai, Peng He, Qilan Cen, Xinyu Luo, Liurong Yang, Angang Ming, Weiwei Shu, Weijun Shen

**Affiliations:** 1 Guangxi Key Laboratory of Forest Ecology and Conservation, College of Forestry, Guangxi University, Nanning, China; 2 Inner Mongolia Environmental Monitoring Station Hohhot Branch, Hohhot, China; 3 Experimental Centre of Tropical Forestry, Chinese Academy of Forestry, Pingxiang, China; 4 Guangxi Youyiguan Forest Ecosystem Research Station, Youyiguan Forest Ecosystem Observation and Research Station of Guangxi, Pingxiang, China

**Keywords:** even-aged mixed plantation, uneven-aged mixed plantation, community composition, co-occurrence network, network complexity, keystone taxa

## Abstract

**Introduction:**

Mixed-species plantations are a promising strategy to increase productivity and promote sustainable forest management. However, the effects of even-aged and uneven-aged tree species mixtures on microbial communities along the root–soil continuum remains underexplored.

**Methods:**

We investigated the bacterial and fungal communities across three distinct microhabitats—bulk soil, rhizosphere soil, and fine roots—within two mixed plantations (even- and uneven-aged) and their respective monocultures.

**Results:**

Our results show that while even-aged mixing had no significant impact on microbial alpha diversity across the three microhabitats, uneven-aged mixing significantly altered some specific indices, indicating that uneven-aged mixing has a greater impact on microbial alpha diversity than even-aged mixing. Both mixing modes significantly altered microbial community composition, with mixed plantations exhibiting intermediate characteristics between monocultures. Although microbial taxonomic and functional compositions were largely unaffected by even-aged and uneven-aged mixing in most cases, distinct differences emerged across microhabitats. Notably, mixed plantations showed increased complexity in fungal co-occurrence networks and harbored more bacterial and fungal keystone species. Fungal communities were more sensitive to both mixing modes, whereas bacterial communities were more strongly influenced by soil environmental factors, particularly pH, which emerged as the primary driver of microbial variation across all plantation types.

**Discussion:**

Our findings highlight that the effects of tree species mixing on microbial communities vary significantly with mixing mode, microhabitat, and microbial taxa, and these should be emphasized in future research and silvicultural practices.

## 1 Introduction

Tree species diversity is known to significantly influence microbial communities via both direct and indirect pathways (Scheibe et al., 2015; Urbanová et al., 2015). Directly, forests with rich species diversity generally provide a diverse and abundant supply of litter and rhizodeposition (e.g., root exudates) to the soil (Prescott and Grayston, 2013; Mori et al., 2020). These inputs can increase resource availability for soil microorganisms, potentially supporting a more diverse and abundant microbial community (Steinauer et al., 2016; Gillespie et al., 2021). For example, Bai et al. (2023b) demonstrated that incorporating broadleaf species into a conifer plantation altered the microbial community involved in litter decomposition. Indirectly, tree species diversity may shape microbial communities by influencing various soil properties (e.g., pH, texture, and nutrient availability) and microclimate (e.g., air temperature, humidity, and light availability), thereby establishing a complex mosaic of microhabitats that promote niche differentiation and specialization among microbes (Tedersoo et al., 2016; Beugnon et al., 2021). A notable example is the increased soil bacterial diversity observed in mixed *Cunninghamia lanceolata* and *Phoebe bournei* plantations, attributed to improved soil nutrient availability (Zhang et al., 2021). Given the critical role of microbial communities in maintaining soil fertility and ecosystem health, it is essential to explore how tree species mixing shapes the microbial community.

The comparison of microbial communities in pure and mixed plantations provides a robust framework for elucidating the impacts of individual tree species and their combinations on these communities (Bai et al., 2023a). Although numerous studies have explored the effects of mixed tree species on soil microbial communities within this framework, the results remain inconclusive. Some studies suggest that tree species mixing generally increases microbial diversity and alters community composition, which may be attributed to improved soil properties such as altered pH (Liu et al., 2010; Zhang et al., 2021), increased organic matter content (Zhang et al., 2021; Li et al., 2021), and enhanced nitrogen and phosphorus availability (Liu et al., 2010; Pereira et al., 2021). Conversely, other studies indicate that mixing tree species may reshape community composition without necessarily increasing diversity (Likulunga et al., 2021; Bai et al., 2023a). Moreover, different microbial taxa or functional groups respond differently to tree species mixing (Ding et al., 2022). For instance, Xu et al. (2023) discovered that fungal communities were predominantly influenced by tree species identity, while bacterial communities were more sensitive to the soil depth. Similarly, the introduction of broadleaf trees into pure pine stands shifted the dominance from saprophytic fungi in pine monocultures to symbiotic fungi in mixed forests (Wu et al., 2019; Li et al., 2021). These findings highlight the complex interactions between trees and soil microbes, resulting in the integration of microbial communities associated with each species in mixed forests (Garau et al., 2019). As a result, further studies are critical to elucidate how the combination of tree species influences microbial community assembly.

There are typically two modes of mixed tree species in forestry practice: even- and uneven-aged mixing. These modes often result in distinct stand structures owing to the different planting times of the target species, which may have different effects on soil microbial communities. Notably, uneven-aged mixed forests sequestered more soil carbon than even-aged forests (You et al., 2018). Xia et al. (2024) found that uneven-aged mixing promoted nutrient acquisition and utilization by *Schima superba* compared to even-aged mixing. These results implied that the influence of tree species mixtures on microbial communities are influenced by the mixing mode. However, most research has focused on the effects of either even- or uneven-aged mixtures on soil microbial communities, and it remains uncertain whether and how these two mixing models influence microbial communities in different directions and magnitudes. Filling this knowledge gap is vital for advancing our understanding of how tree species mixtures shape microbial communities.

Microhabitats within the root–soil continuum, such as bulk soil, rhizosphere, and root endosphere, significantly influence microbial communities due to their unique physicochemical properties (Beckers et al., 2017; Zhou et al., 2022). These microhabitats harbor microbial communities with distinct composition and diversity (Cregger et al., 2018; Trivedi et al., 2020). Notably, microbial diversity in the rhizosphere and root endosphere is often lower than in bulk soil, likely due to reduced selection pressure from the root to the soil (Reinhold-Hurek et al., 2015; Xiong et al., 2021b). Essentially, plants selectively enrich beneficial microbes in their rhizosphere while excluding others, with the bulk soil serving as a reservoir, the rhizosphere as a growth chamber, and the root endosphere as a restricted zone (Van Der Linde et al., 2018; Cordovez et al., 2019). Although these studies have primarily focused on annual grasses and crops, they provide valuable insights into the host selection pressure and environmental filtering gradient in the root–soil continuum. However, current knowledge of microbial community responses to tree species mixtures is largely based on bulk soils, and variation across microhabitats remains poorly understood.

*Pinus massoniana* Lamb., a key timber and afforestation species, is widely cultivated across southern China due to its considerable economic and ecological value (National Forestry and Grassland Administration, 2019). Nonetheless, the long-term establishment of large-scale monoculture plantations of this species has led to several ecological problems that consistent with global monoculture plantations dilemmas, including biodiversity loss, soil degradation, increased susceptibility to pests and diseases, decreased productivity, and weakened ecosystem stability and ecological services (He et al., 2013; You et al., 2018). Converting these monocultures into mixed conifer–broadleaf plantations by replanting precious native broadleaf trees, such as *Castanopsis hystrix*, has been proposed as an effective nature-based silvicultural strategy to address these challenges (Wang et al., 2018; You et al., 2018). This practice is increasingly adopted globally due to its documented benefits, including increased forest productivity (He et al., 2013), accelerated decomposition of recalcitrant conifer litter (Wang et al., 2018), and enhanced carbon sequestration (Wang et al., 2018; You et al., 2018). More critically, mixed-species plantations are increasingly regarded as a cornerstone strategy for improving ecosystem resilience to climate change by maintaining functional diversity and stability under shifting environmental conditions (Schnabel et al., 2021; Chen et al., 2023). The ability of these plantations to maintain stable nutrient cycling and carbon sequestration under environmental stress is largely mediated by soil microbial communities. Therefore, elucidating how tree species mixing shapes microbial assembly and function—as examined in this study—is essential for guiding climate-adaptive forest management strategies that harness plant–microbe interactions. Nonetheless, it remains unclear how microbial communities across different microhabitats respond to specific silvicultural practices, such as even-aged versus uneven-aged mixing regimes.

To bridge these knowledge gaps, we sampled and analyzed bacterial and fungal communities from three distinct microhabitats—bulk soil, rhizosphere, and fine roots—in two mixed plantations (even- and uneven-aged) and their corresponding monospecific plantations. Our objectives were to explore the effects of tree species mixing on microbial alpha diversity, community composition, taxonomic and functional composition, and co-occurrence network complexity. We hypothesized that (H1) both mixing modes would increase microbial alpha diversity, alter community composition, and increase network complexity compared to monocultures; (H2) microbial communities in different microhabitats would respond differently to even- and uneven-aged mixing because these microhabitats are affected differently by root exudates and litter; and (H3) fungal communities would be more susceptible to both mixing modes than bacterial communities due to the strength of their symbiotic associations with plants. This multi-microhabitat research framework offers valuable insights for designing climate-resilient forests worldwide by connecting microbial ecology with global ecological restoration practices.

## 2 Materials and methods

### 2.1 Study site

The study was conducted at the Fubo Experimental Forest Farm (106°51′–106°53′E, 22°02′–22°04′N, 430–680 m above sea level), which belongs to the Experimental Center of Tropical Forestry, Chinese Academy of Forestry, located in Pingxiang City, Guangxi Zhuang Autonomous Region, Southwest China. The region experiences a typical southern subtropical monsoon climate, which is characterized by distinct wet (April–September) and dry (October–March of the following year) seasons. The average annual temperature in the area is 21.7 °C, which ranges from 13.5 °C in January to 27.6 °C in July. The average annual rainfall is 1,376 mm, of which approximately 80% falls during the wet season (April through October). The frost-free period lasts approximately 340 days, and the total annual sunshine amounts to 1,419 h. The potential evaporation is estimated to be between 1,200 and 1,300 mm per year, and the relative humidity varies between 80% and 84%. The soil originates from weathered granite and is classified as a ferrosol according to the Chinese soil classification system, which is equivalent to an oxisol based on the United States Department of Agriculture Soil Taxonomy (IUSS Working Group WRB, 2022). The soil bulk density was 1.23 ± 0.12 g cm^−3^, and the soil texture was dominated by silt (50.24 ± 3.95%), followed by sand (35.83% ± 4.98%) and clay (13.93% ± 3.12%) (Qin et al., 2024). Historically, this area was covered by subtropical evergreen broad-leaved forests, which were cleared in the 1950s to establish monoculture plantations (e.g., *P. massoniana* and *C. lanceolata*). Since the 1980s, some of these plantations have been transformed into mixed conifer-broadleaf plantations by planting native broadleaf species, such as *C. hystrix*, in the understory.

### 2.2 Experimental design

Based on the “triplet” concept (Pretzsch et al., 2015), five representative plantation types were chosen for this study, including (1) a pure *P. massoniana* plantation established in 1983 (PM83), (2) a pure *C. hystrix* plantation planted in 1983 (CH83), (3) an even-aged mixed plantation consisting of *P. massoniana* and *C. hystrix*, both planted in 1983 (EMP), (4) a pure *P. massoniana* plantation created in 1959 (PM59), and (5) an uneven-aged mixed plantation, created by replanting the *C. hystrix* in 1983 under a pure *P. massoniana* plantation that was originally established in 1959 (UMP). Each plantation had trees spaced 2 × 2 m apart, leading to an initial planting density of 2,500 trees per hectare. In the mixed plantations, the two species were arranged in alternating rows at a 1:1 ratio. All plantations were subjected to similar management practices throughout the study. In July 2021, three sampling plots were randomly established for each of the five types of plantations. The detailed stand characteristics for the five plantation types are presented in Supplementary Table S1.

### 2.3 Sample collection and processing

In January 2022, bulk soil, rhizosphere soil, and fine root samples at 0–40 cm depth were collected from the five plantations mentioned above. For fine root and rhizosphere soil sampling, six target trees were selected in each monoculture plantation, while 12 target trees (two species, six per species) were chosen in each mixed plantation. These selected trees had a DBH and height close to the stand average and were spaced at least 5 m from other trees of the same species. Fine roots (diameter < 2 mm) were collected from these target trees using the root tracking method (Han et al., 2022). Briefly, we initially identified coarse roots that could be traced back to their respective parent tree trunks and then dug down along these coarse roots to their terminal branches. Fine roots attached to the coarse roots were collected, gently agitated and the soil firmly attached to their surfaces was collected and defined as rhizosphere soil. For bulk soil samples, five soil cores (5 cm in diameter) were drilled with a cylindrical drill from the location where the fine roots were collected and homogenized into a composite sample. Finally, a total of 30 bulk soil samples, 42 rhizosphere soil samples, and 42 fine root samples were obtained. The obtained samples were temporarily placed in an insulated container filled with dry ice and immediately transported to the laboratory. Upon arrival, roots, stones, and litter were carefully removed from the soil samples, which were then sieved through a 2.0-mm mesh and divided into three subsamples. The first subsample was immediately frozen at −80 °C for DNA extraction; the second was stored at 4 °C for the immediate analysis of soil available nitrogen and enzyme activities; and the third was air-dried at 25 °C for other soil physicochemical analyses. After cleaning and surface sterilization, the fine root samples were frozen at −80 °C until further processing.

### 2.4 Soil chemical analyses

The detailed methods for measuring soil chemical properties have been described by Bai et al. (2023a). Briefly, soil pH was assessed with a pH meter (FE28; Mettler Toledo Instruments Co. Ltd., Shanghai, China) in a soil suspension prepared by mixing air-dried soil with a 1 M KCl solution at a ratio of 1:2.5 (w/v). Soil organic matter (SOM) and total nitrogen (TN) were determined by potassium dichromate oxidation with external heating and the automated Kjeldahl method (KJELTEC™ 8,400; FOSS Quality Assurance Co., Ltd., Hillerød, Denmark), respectively. To determine total phosphorus (TP) and available phosphorus (AP), soil samples were extracted with H_2_SO_4_-HClO_4_ and a mixture of hydrochloric acid and sulfuric acid (0.05 M HCl + 0.025 M H_2_SO_4_), respectively. These extracts were then analyzed using the molybdenum-antimony anti-colorimetric method on a multifunctional microplate reader (Infinite M200 Pro; Tecan, Männedorf, Switzerland), with absorbance readings were taken at 880 nm for TP and 882 nm for AP (Olsen and Sommers, 1982). Additionally, ammonium nitrogen (NH_4_^+^-N) and nitrate nitrogen (NO_3_^−^-N) were extracted with a 0.01 M CaCl_2_ solution and measured using a continuous-flow autoanalyzer (AutoAnalyzer-AA3; Seal Analytical, Norderstedt, Germany). Available nitrogen (AN) was calculated as the sum of the NH_4_^+^-N and NO_3_^−^-N concentrations.

### 2.5 Soil extracellular enzyme activity

We employed the microplate fluorometry method (Bell et al., 2013) to assess the potential activities of five key soil extracellular enzymes. These enzymes comprised α-1,4-glucosidase (AG) and β-1,4-glucosidase (BG) for carbon acquisition, β-1,4-N-acetylglucosaminidase (NAG) for carbon and nitrogen acquisition, leucine aminopeptidase (LAP) for nitrogen acquisition, and acid phosphatase (ACP) for organic phosphorus acquisition. For a comprehensive description of the experimental procedures, please refer to Supplementary materials S1.

### 2.6 DNA extraction, PCR amplification, and high-throughput sequencing

The total genomic DNA was extracted from each sample using the HiPure Soil DNA Kit (Magen Biotechnology Co., Ltd., Guangzhou, China) according to the manufacturer’s guidelines. The concentration and purity of the extracted DNA was assessed using a NanoDrop™ 2000 UV-Vis spectrophotometer (Thermo Fisher Scientific, Wilmington, DE, USA), and its quality further verified through electrophoresis on a 1% (w/v) agarose gel. To profile the bacterial communities, we amplified the V5–V7 region of the 16S rRNA gene utilizing the primer pair 799F and 1193R (Beckers et al., 2017). For the fungal communities, the ITS1 region was targeted for amplification and sequencing with the ITS1-F_KYO2 and ITS86R primers (Scibetta et al., 2018). The raw sequence data for both bacterial and fungal amplicons were deposited in the NCBI Sequence Read Archive, with the corresponding accession numbers PRJNA1176343 and PRJNA1176356, respectively. The detailed procedures for PCR amplification and high-throughput sequencing can be found in Supplementary materials S2.

### 2.7 Bioinformatic analyses

The raw sequencing data were processed using the USEARCH pipeline (version 10.0; Edgar, 2010) and QIIME2 (v2020.11; Caporaso et al., 2010). Initially, sequences containing primer sequences as well as low-quality ends with quality scores below 30 were eliminated from the raw reads. Subsequently, paired-end sequences were merged and trimmed to 200 base pairs, followed by quality filtering with a maximum expected error threshold set at 0.5 using USEARCH. Duplicates and singletons were excluded and non-chimeric sequences were clustered into amplicon sequence variants (ASVs) through DADA2 (version 1.14; Callahan et al., 2016). The Ribosomal Database Project classifier tool (version 2.2) was used for the taxonomic classification of representative bacterial and fungal sequences, utilizing the SILVA (version 132; Quast et al., 2012) and UNITE (version 9.0; Nilsson et al., 2019) databases, respectively. To reduce potential sequencing errors, sequences that occurred only once or in a single sample were omitted from the ASV table. Additionally, to ensure consistency in sequencing depths across samples, all sequencing data were rarefied to the minimum sequencing depth prior to downstream analysis. The determination of bacterial functional guilds was performed using the FAPROTAX database (Louca et al., 2016), whereas the identification of fungal trophic groups was performed using the FUNGuild database (Nguyen et al., 2016). To minimize over-interpretation, only those fungal community functions classified as “highly probable” and “probable” were included in the analysis.

### 2.8 Network construction and analysis

Network analysis was performed at the ASV level to explore how mixing different tree species influences microbial community interactions. We constructed the co-occurrence network using the “SparCC” method available on the iNAP platform (Feng et al., 2022). Initially, we filtered the raw data to retain only those ASVs that were in the top 10% of the total abundance and were present in at least 50% of the samples within each group to avoid spurious correlations. The significance of the correlations between microbial taxa abundances was estimated with 100 bootstrap replicates, and only robust (Spearman’s correlation coefficient |*r*| > 0.6) and statistically significant (*p* < 0.05) correlations were included in the network construction (Steinhauser et al., 2008). To assess the validity of the empirical network, we generated 100 random networks in which all the connections were randomly rearranged (Deng et al., 2012). We analyzed microbial network structure by measuring the number of elements and connections, how interconnected, clustered, and modular they were, and how efficiently information could flow through them. Specifically, we computed various topological properties of these networks, including the number of nodes and edges, average degree, average clustering coefficient, average path distance, modularity, transitivity, connectance, and efficiency. Visualization of these co-occurrence networks was performed using the Gephi interactive platform (version 0.9.2; Bastian et al., 2009). To accurately identify potential keystone taxa within the network, we computed the within-module connectivity (*Zi*) and among-module connectivity (*Pi*) for each node (Guimerà and Nunes Amaral, 2005). The nodes were then divided into four different classifications: network hubs (high connectivity within or across modules, defined by *Zi* > 2.5 and *Pi* > 0.62), module hubs (high within-module connectivity, with *Zi* > 2.5 and *Pi* < 0.62), connectors (high among-module connectivity, characterized by *Zi* < 2.5 and *Pi* > 0.62), and peripherals (lower connectivity, defined by *Zi* < 2.5 and *Pi* < 0.62) (Guimerà and Nunes Amaral, 2005). The nodes identified as network hubs, module hubs, and connectors were classified as keystone nodes (Deng et al., 2012).

### 2.9 Statistical analyses

Before conducting statistical analyses, we assessed the normality and homogeneity of our data using the Shapiro–Wilk and Levene’s tests, respectively. Data were log-transformed if deemed necessary. To assess the differences in soil environmental factors among different plantation types in the same microhabitat or among different microhabitats under the same plantation type, we conducted a one-way ANOVA, followed by Tukey’s *post-hoc* test. We calculated the alpha diversity metrics, including Chao1 and Shannon indices for the microbial communities, at the ASV level utilizing the “diversity” function from the “vegan” R package (Oksanen et al., 2022). To evaluate differences in microbial attributes (e.g., alpha diversity, taxonomic composition, and functional composition) among different plantation types within the same microhabitat or among different microhabitats within the same plantation type, we used nonparametric tests (either Kruskal–Wallis or Wilcoxon tests) facilitated by the “EasyStat” R package. Furthermore, to analyze the interactive effects of the plantation type and microhabitat on the soil environmental factors, microbial alpha diversity, taxonomic composition, and functional composition, a linear mixed-effects model was used. The “lmer” function from the “lme4” R package (Bates et al., 2015) was used in this model, with trees nested within plots considered a random factor. Additionally, we evaluated variations in the microbial community composition among the treatment groups through an abundance-weighted matrix (specifically, Hellinger-transformed Bray–Curtis dissimilarity), with results visualized using NMDS plots generated by the “metaMDS” function within the “vegan” R package. We then analyzed the relative contribution of the plantation type, microhabitats, and their interactions to community dissimilarity using PERMANOVA, accompanied by 999 permutations via the “adonis2” function from the “vegan” R package. To explore the relationship between the soil environmental factors, we conducted pairwise comparisons utilizing Pearson’s correlation analysis. Subsequently, we executed Mantel tests to identify the key factors influencing microbial alpha diversity, community composition, taxonomic composition, and functional composition using the “qcorrplot” function from the “linkET” R package. Redundancy analysis (RDA) was performed using the “rda” function from the “vegan” R package to explore the correlation between the microbial community composition and soil environmental factors. The significance of the RDA results was determined by a Monte Carlo permutation test with 999 permutations, implemented with the “envfit” function in the “vegan” R package (Legendre and Legendre, 2012; Oksanen et al., 2022). All statistical significance was determined at a threshold of *p* < 0.05, unless otherwise specified. All statistic statistical analyses were conducted using R (version 4.3.2).

## 3 Results

### 3.1 Soil environmental variability

The chemical properties and enzyme activities of bulk soil and rhizosphere soil in two mixed plantations and their respective monocultures were shown in Supplementary Table S2. Linear mixed-effects models showed that microhabitat significantly affected almost all soil chemical properties and enzyme activities, whereas plantation type and the interaction between microhabitat and plantation type only significantly affected a small number of soil chemical properties and enzyme activities (Supplementary Table S3). One-way ANOVA indicated that neither even-aged nor uneven-aged mixing significantly affected most bulk soil environmental factors, although their mean values increased (Supplementary Table S2). Both mixing methods significantly decreased the rhizosphere soil pH, while increasing most of the rhizosphere soil chemical properties and enzyme activities such as TN (Supplementary Table S2). Therefore, the influence of even- and uneven-aged mixing on the chemical properties and enzyme activities of the rhizosphere soil was more pronounced than in the bulk soil. Furthermore, the rhizosphere soil generally had a significantly lower pH than the bulk soil across most plantation types, whereas chemical properties including SOM, TN, TP, NO_3_^−^-N, AN, and enzyme activities including those of AG, BG, and NAG were significantly higher than in the bulk soil (Supplementary Table S2).

### 3.2 Microbial diversity

**Figure 1:**
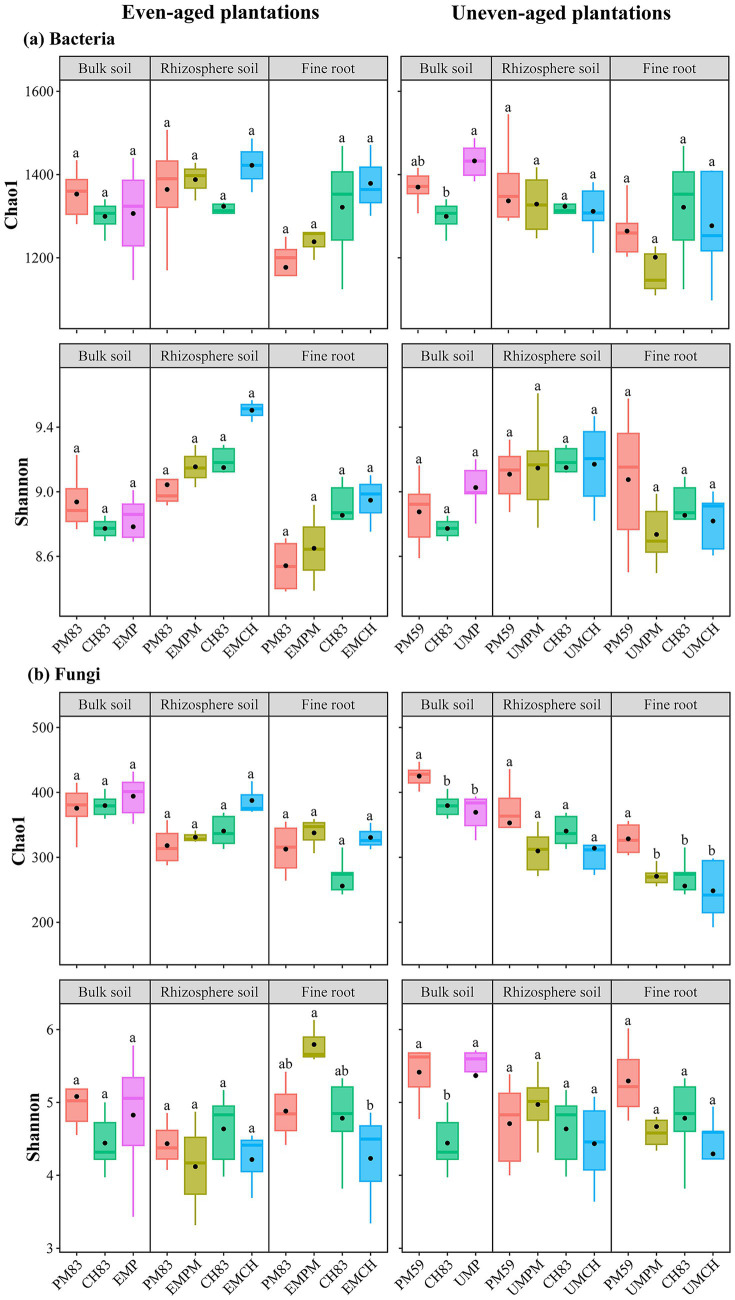
Alpha diversity of bacterial **(a)** and fungal **(b)** communities across different microhabitats (i.e., bulk soil, rhizosphere soil, and fine root) in two mixed plantations and their respective monocultures. The lower and upper boundaries of each box represent the 25th and 75th quartiles, respectively. The whiskers in each box represent 1.5 times the interquartile range, and the horizontal lines and dots inside the boxes indicate the median and mean values, respectively. Different lowercase letters above the error bars indicate significant differences (*p* < 0.05) among plantation types, as determined by the non-parametric Kruskal–Wallis test. PM83, pure *P. massoniana* plantation planted in 1983; CH83, pure *C. hystrix* plantation planted in 1983; EMP, even-aged mixed *P. massoniana* and *C. hystrix* plantation; EMPM, *P. massoniana* in the even-aged mixed *P. massoniana* and *C. hystrix* plantation; EMCH, *C. hystrix* in the even-aged mixed *P. massoniana* and *C. hystrix* plantation; PM59, pure *P. massoniana* plantation planted in 1959; UMP, uneven-aged mixed *P. massoniana* and *C. hystrix* plantation; UMPM, *P. massoniana* in the uneven-aged mixed *P. massoniana* and *C. hystrix* plantation; UMCH, *C. hystrix* in the uneven-aged mixed *P. massoniana* and *C. hystrix* plantation.

Overall, neither even-age nor uneven-age mixing caused a statistically significant change in alpha diversity for the bacterial (Figure 1a) and fungal (Figure 1b) communities, although the means showed an increasing trend (Supplementary Table S4). Consistent with this, a linear mixed-effects model indicated that microhabitats significantly influence the alpha diversity of bacterial and fungal communities, while plantation type do not significantly affect the alpha diversity of either bacterial or fungal communities (Supplementary Table S5). Furthermore, only a few alpha diversity indices were significantly affected by the interaction between plantation type and microhabitat (Supplementary Table S5). Notably, even-aged mixing had no significant effect on the microbial diversity across the three microhabitats (Figure 1). However, uneven-aged mixing significantly reduced the fungal Chao1 index in the bulk and fine roots of *P. massoniana*, whereas it significantly increased the bacterial Chao1 index and fungal Shannon index in the bulk soil of *C. hystrix* (Figure 1). These results suggest that uneven-aged mixing has a greater impact on microbial diversity than even-aged mixing, and the effects of both mixing methods were more pronounced on fungal diversity than on bacterial diversity. Furthermore, microbial alpha diversity was significantly higher in bulk and rhizosphere soils than in fine roots (Supplementary Table S4).

**Figure 2:**
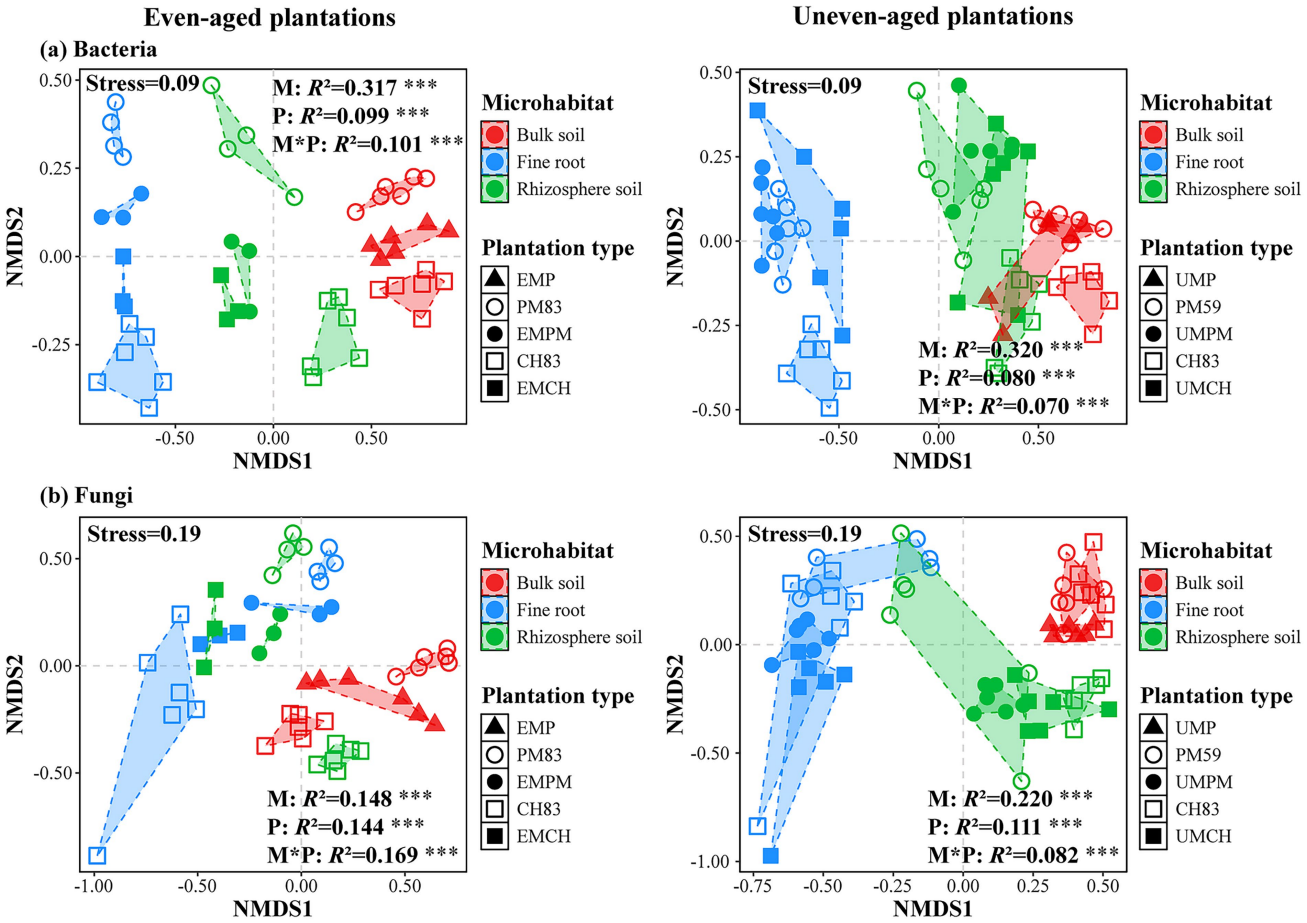
Non-metric multidimensional scaling (NMDS) ordinations and permutational multivariate analysis of variance (PERMANOVA) based on Bray–Curtis distance matrices illustrating the distribution patterns of bacterial **(a)** and fungal **(b)** communities across different microhabitats in two mixed plantations and their respective monocultures. The statistical significance and relative contribution of plantation type, microhabitats, and their interactions to community dissimilarity were assessed by nested PERMANOVA. *p < 0.05, **p < 0.01, and ***p < 0.001. “M” represents the effect of microhabitat, and “P” represents the effect of plantation type. The abbreviations for each plantation type are consistent with those used in Figure 1.

NMDS analysis clearly showed that microbial communities, both bacterial (Figure 2a) and fungal (Figure 2b), exhibited significant differences among different plantation types and microhabitats (*p* < 0.001). Specifically, rhizosphere microbial communities were intermediate between those in bulk soil and fine roots in almost all plantations. Similarly, within each microhabitat, communities in mixed stands were intermediate to those in monocultures. PERMANOVA results further revealed that the microhabitat was the primary factor driving bacterial community variability, followed by the plantation types and their interactions (Figure 2a). Conversely, in even-aged plantations, changes in the fungal communities were influenced by the microhabitats, plantation type, and their interactions, whereas in uneven-aged plantations, microhabitats were the main factor (Figure 2b). Moreover, the fungal community composition was more profoundly influenced by the plantation type than the bacterial community composition.

### 3.3 Microbial taxonomic and functional composition

**Figure 3:**
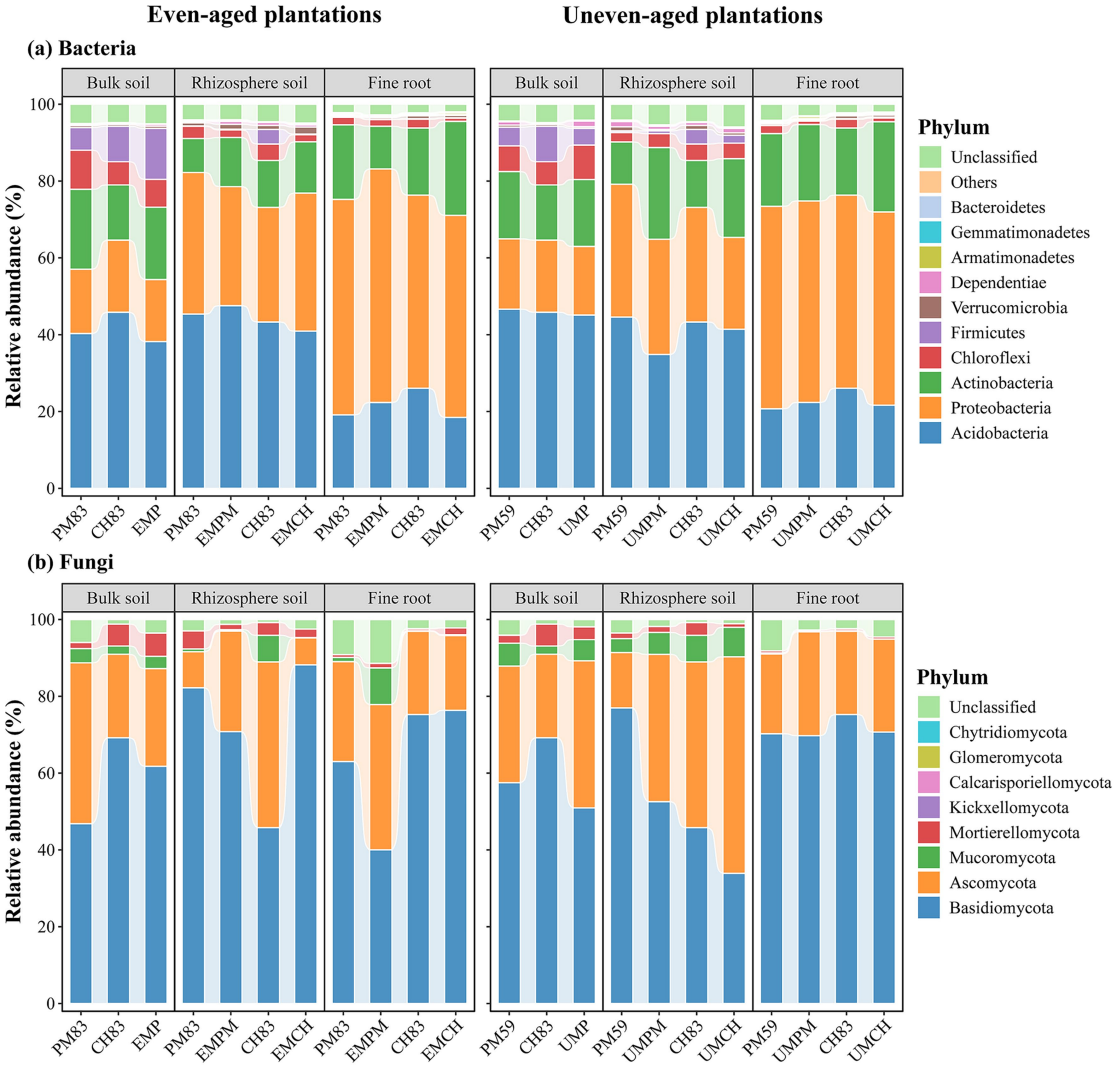
Taxonomic composition of bacterial **(a)** and fungal **(b)** communities across different microhabitats in two mixed plantations and their respective monocultures. The relative abundance of bacteria and fungi at the phylum level was categorized based on the proportional frequencies of the DNA sequences. The abbreviations for each plantation type are detailed in Figure 1.

Across all the plantation types and microhabitats, the most abundant bacterial phyla were Acidobacteria (19.14%–47.57%), Proteobacteria (16.17%–60.81%), and Actinobacteria (8.83%–24.46%), which together accounted for over 73% of the total abundance (Figure 3a; Supplementary Table S6). Linear mixed-effects models revealed that microhabitat significantly affected the relative abundance of almost all bacterial phyla, whereas the relative abundance of only a few bacterial phyla were significantly influenced by plantation type and the interaction between microhabitat and plantation type (Supplementary Table S7). Non-parametric tests indicated that neither even-aged nor uneven-aged mixing had a significant influence on the relative abundance of most bacterial phyla, except for a few phyla that exhibited significant differences (Supplementary Table S6). However, the relative abundance of certain phyla differed significantly across microhabitats. For example, Proteobacteria increased significantly and Acidobacteria decreased significantly from bulk and rhizosphere soils to fine roots (Figure 3b; Supplementary Table S6).

The most abundant fungal phyla were Basidiomycota (33.90%–88.18%) and Ascomycota (7.04%–56.38%) across all the plantation types and microhabitats, which together accounted for more than 77% of the total abundance (Figure 3b; Supplementary Table S8). Linear mixed-effects models revealed that microhabitat and the interaction between plantation type and microhabitat significantly affected the relative abundance of most fungal phyla, whereas plantation type had no significant effect on the relative abundance of all fungal phyla (Supplementary Table S9). Non-parametric analyses revealed that neither even-aged nor uneven-aged mixing significantly influenced the relative abundance of most bacterial phyla (Supplementary Table S8). Similarly, most fungal phyla did not exhibit significant differences in relative abundance across the microhabitats, except for a few that did (Figure 3b; Supplementary Table S8). Additionally, the differences in fungal abundance at the phylum level were more pronounced among various plantation types compared to bacteria, suggesting that the fungal community composition was more responsive to the plantation type (Figure 3; Supplementary Tables S6, S8).

**Figure 4:**
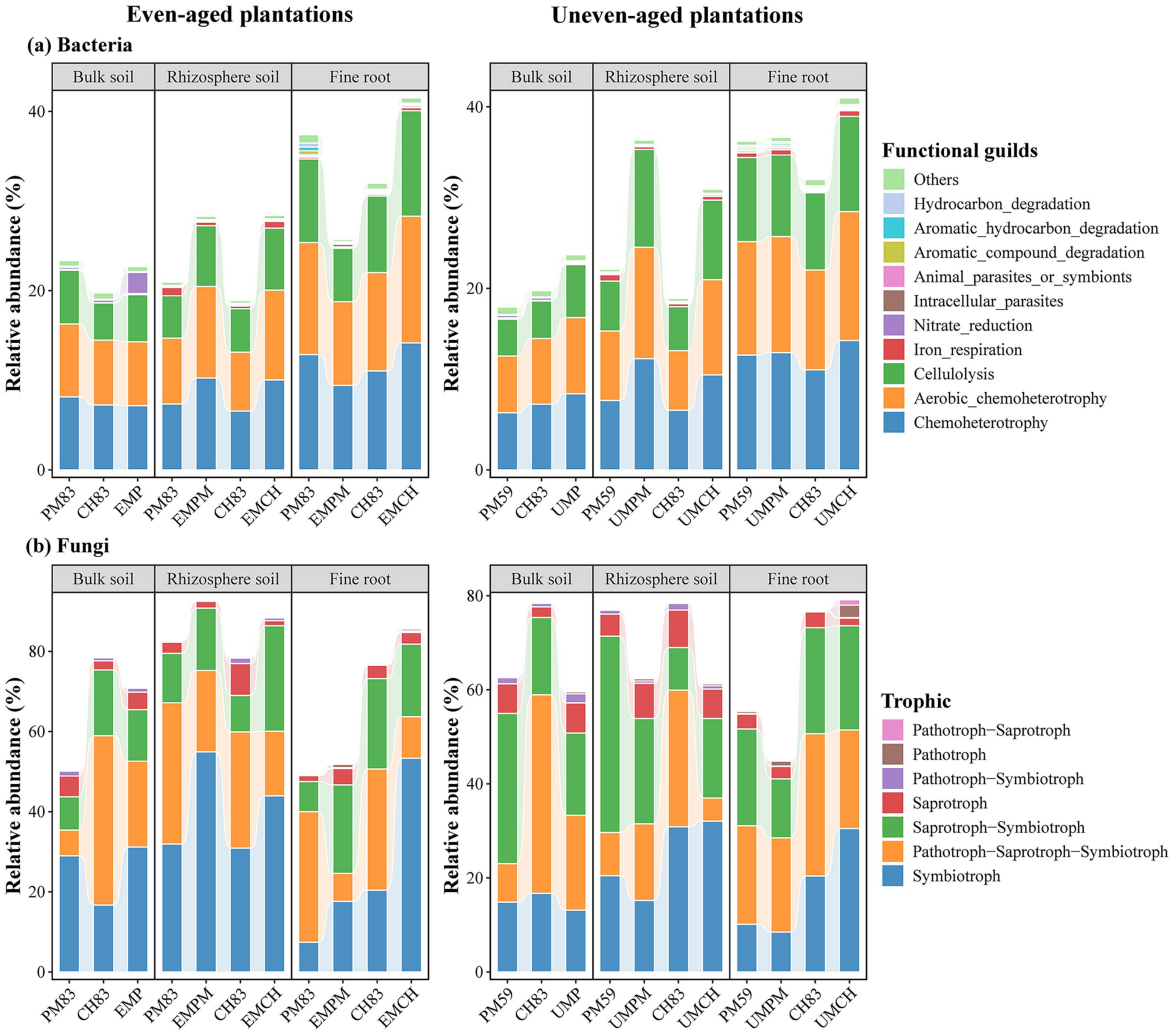
Functional composition of bacterial **(a)** and fungal **(b)** communities across different microhabitats in two mixed plantations and their respective monocultures. The relative abundance of each bacterial functional guild and fungal trophic group was predicted based on the “FAPROTAX” and “FUNGuild” databases, respectively. The figure does not illustrate the relative abundance of unassigned functional groups, which corresponds to the portion of the figure that is not shown. The abbreviations for each plantation type are consistent with those used in Figure 1.

Bacterial function prediction revealed that the most abundant functional guilds were Chemoheterotrophy (6.29%–14.26%), Aerobic-chemoheterotrophy (6.26%–14.20%), and Cellulolysis (4.07%–11.76%) (Figure 4a; Supplementary Table S10). Linear mixed-effects models confirmed that microhabitat significantly affected the relative abundance of all bacterial functional guilds, whereas plantation type did not significantly affect their relative abundance (Supplementary Table S11). Furthermore, only a few bacterial functional guilds were significantly influenced by the interaction between plantation type and microhabitat (Supplementary Table S11). Non-parametric tests showed that the relative abundance of most bacterial functional groups was not affected by even- and uneven-aged mixing practices (Supplementary Table S10). Nevertheless, certain bacterial functional guilds exhibited notable variations in their relative abundance across microhabitats, particularly Chemoheterotrophy and Aerobic-chemoheterotrophy, which increased significantly from bulk and rhizosphere soils to fine roots (Figure 4a; Supplementary Table S10).

Fungal functional prediction indicated that the predominant trophic groups were Symbiotroph (7.41%–54.92%), Pathotroph-saprotroph-symbiotroph (4.92%–42.22%), and Saprotroph-symbiotroph (0.18%–31.94%) (Figure 4b; Supplementary Table S12). Linear mixed-effects models revealed that microhabitat and their interaction with plantation type significantly influenced the relative abundance of numerous fungal functional guilds, while plantation type did not significantly affect the relative abundance of any fungal functional guilds (Supplementary Table S13). According to nonparametric tests, the relative abundance of the fungal functional groups was unaffected by even- or uneven-aged mixing practices (Supplementary Table S12). While most fungal functional groups displayed consistent relative abundances across microhabitats, a few exceptions were noted (Figure 4b; Supplementary Table S12). Moreover, most fungal functional groups exhibited greater relative abundance variation between plantation types than the bacterial functional groups (Figure 4; Supplementary Tables S10, S12), suggesting that the fungal community composition was more sensitive to the plantation type.

### 3.4 Microbial network structure

**Figure 5:**
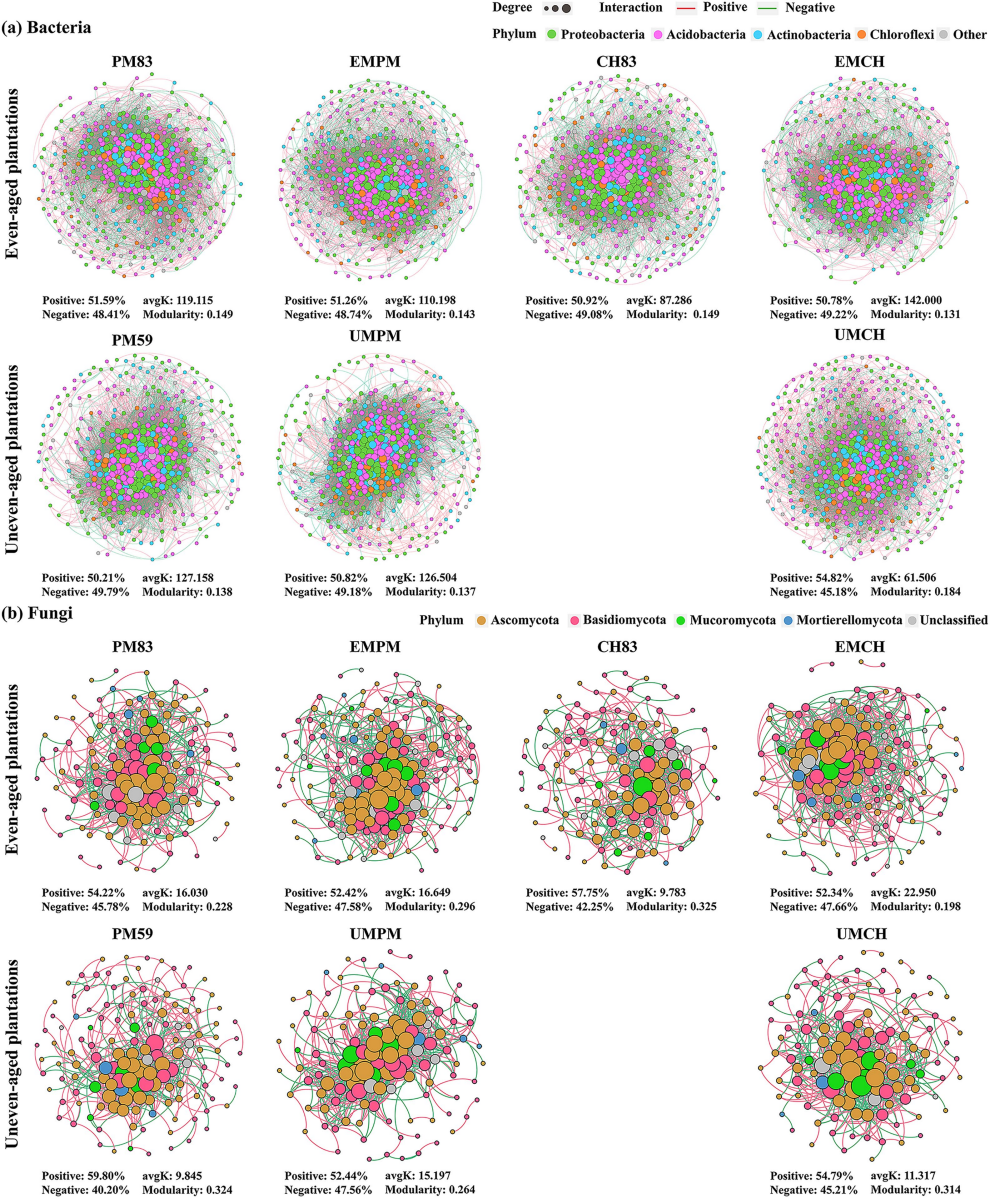
Co-occurrence networks of bacterial **(a)** and fungal **(b)** communities in two mixed plantations and their respective monocultures. Nodes indicate individual amplicon sequence variants (ASVs), which are colored according to the phylum to which they belong, and the size of each node is proportional to its degree of connectivity. Edges represent strong (Spearman’s correlation coefficient |*r*| > 0.6) and statistically significant (*p* < 0.05) correlations between ASVs, with red and green edges representing positive and negative relationships, respectively. “avgK” shows the average degree of the network, whereas “Modularity” indicates the degree to which nodes tend to differentiate into different network modules. The abbreviations for each plantation type are consistent with those used in Table 1. The bacterial and fungal co-occurrence networks of CH83 (blank spaces) in the uneven-aged plantations (lower row) are the same as the networks in the even-aged plantations (upper row).

The structures and topological characteristics of microbial co-occurrence networks varied distinctly across different types of plantations for both bacteria and fungi (Figure 5; Table 1). Specifically, even-aged mixing resulted in fewer nodes, edges, positive edges, and a lower average degree within the bacterial network of *P. massoniana*, indicating a decrease in network complexity and potentially increased competition within bacterial communities (Figure 5a; Table 1). Similarly, the average degree of the *P. massoniana* bacterial network was slightly reduced by uneven-aged mixing, suggesting a slight decrease in complexity (Figure 5a; Table 1). Conversely, even-aged mixing significantly increased the number of nodes, edges, negative edges, and the average degree within the bacterial network of *C. hystrix*, indicating an increase in network complexity and potentially increased competition within the bacterial communities (Figure 5a; Table 1). Uneven-aged mixing led to a decrease in the number of edges, negative edges, and the average degree of the bacterial co-occurrence network of *C. hystrix*, indicating a decrease in network complexity and potentially increased cooperation within bacterial communities (Figure 5a; Table 1). Additionally, both mixing methods led to more nodes, edges, negative edges, and higher average degrees in the fungal networks for both *P. massoniana* and *C. hystrix*, suggesting an increase in network complexity and potentially increased competition within the fungal communities (Figure 5b; Table 1). Compared to the fungal networks, the bacterial networks had more nodes, edges, a higher average degree, and a higher proportion of negative correlations, indicating a higher network complexity for the bacteria but potentially more competition within the bacterial community (Figure 5; Table 1).

**Table 1:** Topological properties of bacterial and fungal co-occurrence networks in each plantation.

Microhabitat	Number of nodes	Number of edges	Positive edges (%)	Negative edges (%)	Average degree (avgK)	Average clustering coefficient (avgCC)	Average path distance (GD)	Modularity	Transitivity (Trans)	Connectance (Con)	Efficiency	Hub nodes		
												Connector hubs	Module hubs	Network hubs
Bacteria even-aged plantations														
PM83	504	30,017	51.59	48.41	119.115	0.636	2.087	0.149	0.692	1.000	0.765	6	0	1
EMPM	496	27,329	51.26	48.74	110.198	0.621	2.123	0.143	0.672	1.000	0.775	20	0	0
CH83	454	19,814	50.92	49.08	87.286	0.600	2.285	0.149	0.657	0.982	0.806	7	1	0
EMCH	489	34,719	50.78	49.22	142.000	0.660	1.959	0.131	0.697	1.000	0.710	11	1	0
Bacteria uneven-aged plantations														
PM59	487	30,963	50.21	49.79	127.158	0.666	2.127	0.138	0.707	0.992	0.738	6	0	0
UMPM	492	31,120	50.82	49.18	126.504	0.680	2.115	0.137	0.695	0.984	0.741	4	0	0
CH83	454	19,814	50.92	49.08	87.286	0.600	2.285	0.149	0.657	0.982	0.806	7	1	0
UMCH	498	15,315	54.82	45.18	61.506	0.528	2.418	0.184	0.559	0.984	0.876	11	1	0
Fungi even-aged plantations														
PM83	133	1,066	54.22	45.78	16.030	0.454	2.486	0.228	0.531	0.912	0.874	9	2	1
EMPM	154	1,282	52.42	47.58	16.649	0.422	2.651	0.296	0.541	0.974	0.894	21	0	0
CH83	120	587	57.75	42.25	9.783	0.402	3.119	0.325	0.479	0.934	0.920	4	0	0
EMCH	160	1836	52.34	47.66	22.950	0.489	2.552	0.198	0.639	0.951	0.854	14	0	0
Fungi uneven-aged plantations														
PM59	142	699	59.80	40.20	9.845	0.379	3.30	0.324	0.521	0.972	0.935	10	2	1
UMPM	127	965	52.44	47.56	15.197	0.467	2.767	0.264	0.586	0.923	0.877	21	0	0
CH83	120	587	57.55	42.25	9.783	0.402	3.119	0.325	0.479	0.934	0.920	4	0	0
UMCH	120	679	54.79	45.21	11.317	0.426	2.728	0.314	0.498	0.903	0.903	14	0	0

The abbreviations for each plantation type are the same as in Figure 1.

The structures and topological features of the microbial co-occurrence networks varied distinctly across different microhabitats for both bacteria and fungi (Supplementary Figure S2; Supplementary Table S14). Specifically, in even-aged plantations, the number of nodes, edges, negative edges, and the average degree of bacterial networks decreased progressively from rhizosphere soils to fine roots and then to bulk soils (Supplementary Figure S2a; Supplementary Table S14). However, in uneven-aged plantations, the number of nodes, edges, positive edges, and the average degree of bacterial co-occurrence networks showed a decreasing trend from bulk soils to rhizosphere soils and then to fine roots (Supplementary Figure S2a; Supplementary Table S14). Moreover, in both types of plantations, fungal networks showed a decrease in nodes, edges, negative connections, and the average degree from rhizosphere soils to bulk soils, then to fine roots (Supplementary Figure S2b; Supplementary Table S14). The bacterial networks had more nodes, edges, higher average degrees, and a greater proportion of positively correlated edges compared to the fungal networks (Supplementary Figure S2; Supplementary Table S14).

Distinct keystone taxa were found in the co-occurrence networks of different plantation types (Supplementary Figure S3). Specifically, even-aged mixing resulted in an increase in keystone species within the bacterial network of *P. massoniana* while uneven-aged mixing resulted in a decrease (Supplementary Figure S3a; Table 1). Conversely, both mixing methods increased the keystone species within the bacterial network of *C. hystrix* (Supplementary Figure S3a; Table 1). Additionally, the keystone species in the fungal networks of *P. massoniana* and *C. hystrix* were increased by both mixing methods (Supplementary Figure S3b; Table 1). Notably, fungal networks contained more keystone species than bacterial networks (Supplementary Figure S3; Table 1).

Further analysis revealed significant differences in the keystone species present within the bacterial and fungal networks across the microhabitats (Supplementary Figure S4). Specifically, a significant decrease in the keystone species was observed in the even-aged plantations from fine roots to bulk soils and then to rhizosphere soils (Supplementary Figure S4a; Supplementary Table S14). Conversely, the keystone species decreased from bulk soil to rhizosphere soil, followed by fine roots, in the bacterial networks of uneven-aged plantations (Supplementary Figure S4a; Supplementary Table S14). Additionally, in both plantations types, the fungal network showed a reduction in the keystone species starting from fine roots, then rhizosphere soil, and finally bulk soil (Supplementary Figure S4b; Supplementary Table S14). Notably, the bacterial network consistently exhibited more keystone species than the fungal network across all microhabitats (Supplementary Figure S4; Supplementary Table S14).

### 3.5 Key drivers of microbial community assembly

**Figure 6:**
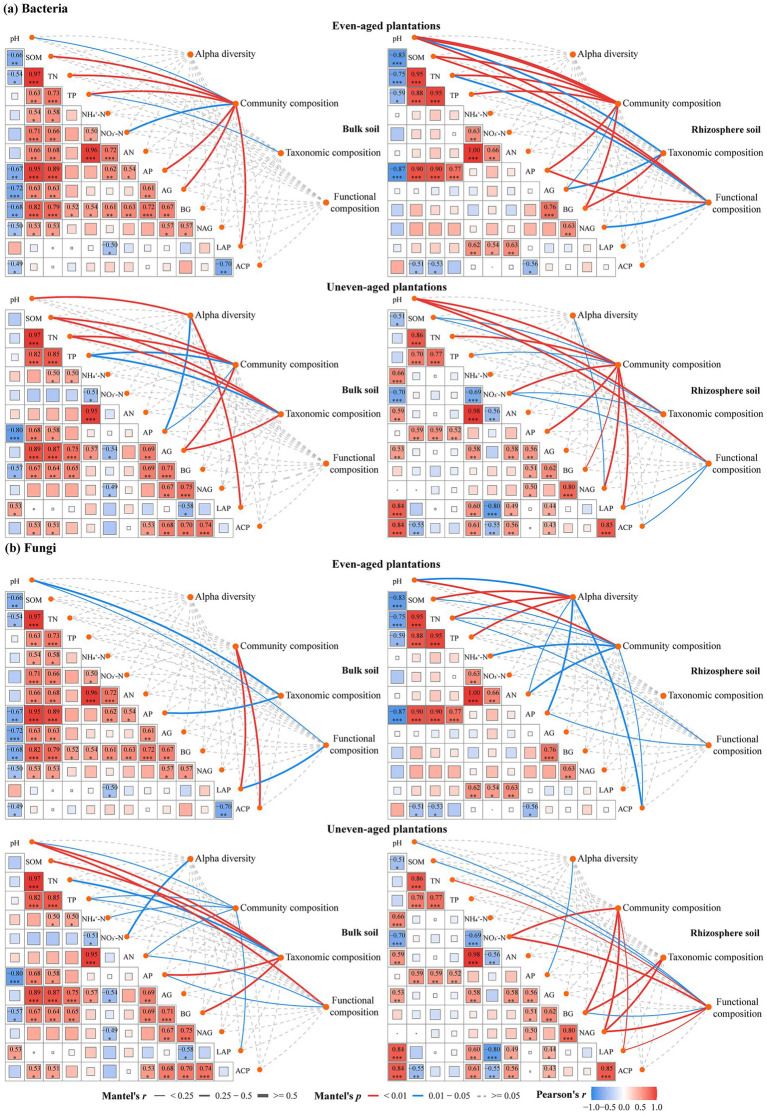
Pearson’s correlation analysis between different soil properties, and Mantel test analysis of bacterial **(a)** and fungal **(b)** alpha diversity, community structure, taxonomic composition, functional composition, and soil properties. The dissimilarity of microbial and soil properties was assessed using the Bray–Curtis distance and Euclidean distance, respectively. Pairwise correlations between soil properties were visualized using a correlation heatmap, where the color gradient indicates the Pearson’s correlation coefficient (ranging from dark blue to dark red, representing a correlation coefficient from −1 to 1) and the size of the box is proportional to the Pearson’s correlation coefficient. Significant correlations are marked with Pearson’s coefficient and significance markers (*p < 0.05, **p < 0.01, and ***p  < 0.001). The width of the edges represents the Mantel’s *r* value for the corresponding distance correlations, and the color of the edges denotes the statistical significance determined by 999 permutations (Mantel’s *p*). Gray dashed edges indicate that microbial indicators do not exhibit a significant correlation with soil properties.

**Figure 7:**
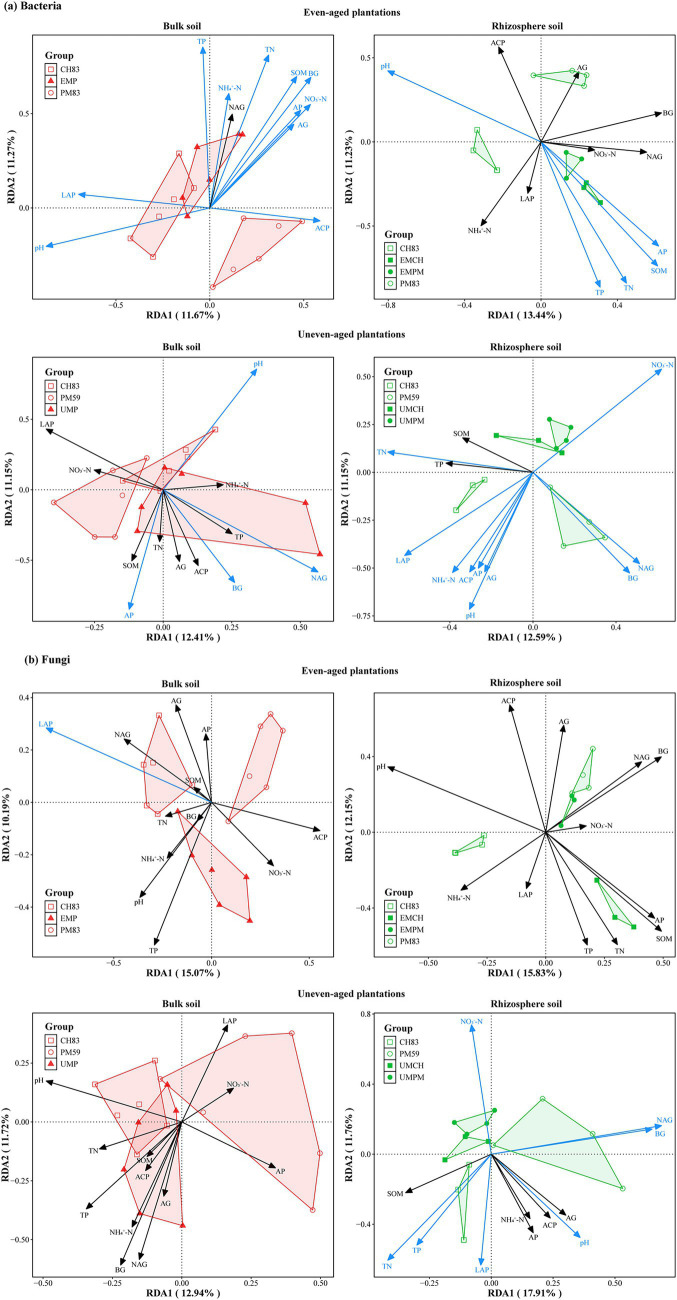
Redundancy analysis (RDA) revealed the effects of soil chemistry and enzyme activities (arrows) on bacterial **(a)** and fungal **(b)** community composition at the ASVs level. Blue arrows indicate soil chemical properties and enzyme activities that had significant effects on microbial community composition (*p* < 0.05).

Mantel tests and RDA revealed that the soil environmental factors influencing the alpha diversity, community composition, taxonomic composition, and functional composition of microbial communities varied with the mixing mode and microhabitat (Figures 6, 7). Notably, the alpha diversity, taxonomic composition, and functional composition of the microbial communities were significantly correlated with only a few soil environmental factors, such as pH and SOM (Figure 6). Overall, the relationships between the microbial communities and soil factors were more pronounced in uneven-aged plantations compared to the even-aged ones. Moreover, these correlations were stronger in the rhizosphere than in bulk soil. Furthermore, the bacterial and fungal community compositions were shaped by different soil environmental factors, with the bacterial community demonstrating greater sensitivity to these factors than the fungal community.

## 4 Discussion

### 4.1 Influence of even- and uneven-aged mixing on microbial communities

Contrary to our first hypothesis, we found that both even- and uneven-aged mixing did not significantly affect microbial alpha diversity in most cases (Figure 1). This result aligns with some previous studies (Bai et al., 2023a; Ding et al., 2023a), but contrasts with others (Pereira et al., 2021; Xu et al., 2023; Li et al., 2023), which had demonstrated a notable increase in microbial diversity due to tree species mixing. The inconsistency between these findings can be explained by several factors. First, our recent research indicates that the mixed planting of *P. massoniana* and *C. hystrix* had no significant effect on the rate of root exudate release for either species (He et al., 2025). Second, Wang et al. (2018) found that mixed planting of these two species significantly inhibited the decomposition of *C. hystrix* litter. Finally, this study found that neither even-aged nor uneven-aged mixing significantly altered most soil chemical properties or enzyme activities. Specifically, the Mantel test revealed that there was no significant relationship between microbial alpha diversity and almost all the soil environmental factors (Figure 6). Previous studies, such as those by Li et al. (2021), showed that mixed planting of *P. massoniana* (an ectomycorrhizal species) with arbuscular mycorrhizal tree species significantly increased microbial diversity. However, our study found that the mixing of two ectomycorrhizal species did not significantly increase microbial diversity. This implies that the presence of tree species with different mycorrhizal combinations contributes to increased microbial diversity, as plants with distinct mycorrhizal types tend to recruit a more diverse array of microbial species.

Although even- and uneven-aged mixing did not significantly influence microbial alpha diversity, these strategies substantially changed the microbial communities composition, with communities in mixed plantations intermediate between those observed in monoculture plantations (Figure 2). This observation aligns with Pereira et al. (2021) and Santana et al. (2021), which showed that mixed plantations tend to merge the microbial communities from their respective monocultures. This intermediacy may be explained by the distinct root exudates and litter qualities associated with different tree species, which influence soil nutrient availability and subsequently modify the microbial community composition (Gillespie et al., 2021; Huo et al., 2023). Our recently published research supports this perspective, with results indicating that incorporating *C. hystrix* into *P. massoniana* monocultures significantly alters the root exudate composition of both species (He et al., 2025). Furthermore, our Mantel test and RDA results revealed significant correlations between bacterial community composition and most soil environmental factors. These results suggest that mixing tree species can reshape the soil bacterial community by modifying soil environmental factors. Together, these findings demonstrate that mixed plantations can promote the integration of microbial taxa associated with different tree species, which can have a significant impact on ecosystem functioning.

Network analysis is recognized as an effective approach to infer potential interactions among various microbial taxa (Faust and Raes, 2012; Guseva et al., 2022). Through this approach, we found that both even- and uneven-aged mixing led to a decrease in the bacterial network complexity for *P. massoniana*. Additionally, even-aged mixing significantly increased the bacterial network complexity for *C. hystrix*, whereas uneven-aged mixing decreased it. These results contrast with many previous observations that mixed tree species increase bacterial network complexity (Ding et al., 2022; Ding et al., 2023b). This discrepancy may be related to the growth performance of the species in these two mixed plantations. A recent study conducted at our study site showed that even-aged mixing of these species inhibited the growth of *P. massoniana* but promoted that of *C. hystrix*, whereas uneven-aged mixing suppressed the growth of both species (Chen et al., 2024). This suggests a strong relationship between tree growth performance and bacterial network complexity, likely because growth performance affects the amount and composition of litter and root exudates, which, in turn, shape bacterial community composition and interactions (Baldrian, 2017; Gillespie et al., 2021). Contrary to the bacterial networks, we found that both even- and uneven-aged mixing increased the complexity of the fungal networks associated with *P. massoniana* and *C. hystrix*, and increased competition within these fungal communities. This finding is consistent with the results reported by Ding et al. (2022) and Pereira et al. (2021), and may stem from the ectomycorrhizal properties of the two tree species, which increase interspecific interactions when planted together. Such interactions complicate the fungal co-occurrence network and increase competition within the community. These findings underscore the importance of considering species-specific traits and mixing strategies when establishing mixed-species plantations. Future research should focus on uncovering the mechanisms that govern microbial co-occurrence network complexity, as this knowledge could facilitate manipulating microorganisms to promote sustainable plantation management.

Our research revealed that most mixed plantations displayed a greater abundance of keystone species within their microbial co-occurrence networks (Supplementary Figure S3; Table 1), which is consistent with many studies (Ding et al., 2023a; Ding et al., 2023b; Li et al., 2023). This may be because mixed plantations create a more complex and diverse microhabitats, which could enhance interspecific interactions and increase the prevalence of keystone species (Ding et al., 2023b). Interestingly, the abundance pattern of keystone species was similar to that of network complexity, suggesting that the complexity of microbial interactions is closely linked to the presence of keystone species. This indicated that these keystone species play pivotal roles in facilitating a wide range of interactions and functions within the community (Banerjee et al., 2018). Furthermore, keystone species were found to be more prevalent in fungal networks compared to bacterial networks, especially those belonging to Ascomycetes and Basidiomycetes, emphasizing their significant functional role in ecosystem processes such as decomposition and nutrient cycling. These findings suggest that fungal communities were more sensitive to changes in the stand type, which could have significant implications for forest management practices. Nevertheless, it must be acknowledged that the keystone species identified through network analysis may only be statistically significant (Banerjee et al., 2018; Zheng et al., 2021). Therefore, further experimental studies involving synthetic communities are necessary to validate the ecological roles and importance of these keystone species.

### 4.2 Effects of even-aged and uneven-aged mixing on microbial community vary with the microhabitat

Microhabitats along the root–soil continuum are characterized by unique physicochemical and biological properties that create specialized niches that influence the structure and function of microbial communities (Zhou et al., 2022; Zhong et al., 2022). Our results showed that there are significant differences in the microbial alpha diversity and community composition among different microhabitats (Figures 1–4). Such differences may be caused by the process of microbial colonization in plants (Bulgarelli et al., 2013). Typically, soil acts as a primary reservoir for plant-associated microbiomes, which are first attracted to the rhizosphere and then to the plant roots (Compant et al., 2010). As microbes move from bulk soil and the rhizosphere to the plant roots, host selective pressures increase, leading to the enrichment of certain microbial taxa and the exclusion of others (Vandenkoornhuyse et al., 2015; Xiong et al., 2021b). Consequently, microbial diversity in the fine roots was lower than that in the bulk and rhizosphere soils, and the species composition varied significantly among different microhabitats.

In alignment with our second hypothesis, the effect of mixing even- and uneven-aged tree species on microbial communities was observed to be microhabitat dependent, indicating that these effects are microhabitat specific. This may be due to the significant differences in the physicochemical and biological properties of different microhabitats (Zhou et al., 2022; Zhong et al., 2022). Such differences may create unique environments for microorganisms, thereby selectively recruiting specific microbial communities. Additionally, tree species mixing alters the characteristics of these microhabitats by modifying the amount and composition of litter and root exudates, thereby exerting selective pressures on the microbial populations. This, in turn, leads to different microbial responses to tree species mixing in different microhabitats.

Furthermore, our results revealed that microbial communities exhibited greater sensitivity to microhabitats than to plantation types, with respect to various microbial characteristics, such as alpha diversity, community composition, taxonomic composition, functional composition, and microbial co-occurrence networks, regardless of the mixing mode and microbial taxa involved (Figures 1–5). This aligns with previous research highlighting the critical role of microhabitats in shaping the composition and function of microbial communities within the root–soil continuum (Beckers et al., 2017; Cregger et al., 2018; Xiong et al., 2021a; Xiong et al., 2021b; Zhong et al., 2022). This may be due to the distinct physicochemical properties of different microhabitats in the root–soil continuum, which facilitate the recruitment of specialized microbial taxa (Beckers et al., 2017; Zhong et al., 2022). These findings underscore the importance of considering microhabitat specificity when examining the response of microbial communities to tree species mixing. Future studies should delve into the mechanisms driving these microhabitat-specific effects and examine their potential implications for forest management and conservation practices.

### 4.3 Fungal community was more sensitive to even-aged and uneven-aged mixing than bacterial community

Consistent with our third hypothesis, the fungal community exhibited greater sensitivity to the mixing of even- and uneven-aged tree species than the bacterial community (Figures 1–4), which is aligned with a number of studies (e.g., Li et al., 2021; Xu et al., 2023). Moreover, Mantel test and RDA revealed that bacterial communities have a stronger correlation with soil environmental factors compared to fungal communities (Figure 6), aligning with previous studies (e.g., Xue et al., 2022; De Vries et al., 2018). These differences likely arise from the distinct life history strategies and niche differentiation between fungi and bacteria. Fungi, especially mycorrhizal fungi, often form symbiotic relationships with specific plant species, functioning as rhizosphere symbionts, endophytes, and pathogens, and frequently co-evolve with their host plants (Peay et al., 2013; Gao et al., 2013). In contrast, bacteria generally exhibit less direct association with plant roots (Vos et al., 2013). Additionally, fungi, as the primary decomposers of plant residues, typically decompose recalcitrant organic substances like lignin and cellulose (Broeckling et al., 2008; Crowther et al., 2019). Therefore, fungi are more directly dependent on the habitat and substrates provided by plants, including litter (Broeckling et al., 2008; Urbanová et al., 2015; Van Der Linde et al., 2018). In contrary, bacteria, known for their rapid growth and sensitivity to soil environmental changes, primarily consume easily degradable substances, such as water-soluble sugars and phenolic compounds released during litter decomposition (Urbanová et al., 2015; Banerjee and Van Der Heijden, 2023). Thus, our results suggest that fungi are more strongly affected by the mixing of even- and uneven-aged tree species, while bacterial community composition are more responsive to changes in the soil environment.

### 4.4 Limitations and implications

To our knowledge, this is the first study to simultaneously examine the effects of even- and uneven-aged tree species mixtures on microbial communities within different microhabitats. However, we acknowledge the inherent limitations of our experimental design. First, our research was confined to pure and mixed stands of only two common subtropical reforestation tree species. Given the species-specificity of microbial communities, future studies should explore a broader array of tree species to identify universal mechanisms underlying microbial community responses to tree species mixing. Second, our study primarily focused on the influence of soil environmental factors on microbial communities, without exploring how tree species mixing affects microbial communities through changes in the quantity and quality of root exudates and litter. Therefore, further research should quantify the relative contributions of soil properties, root exudates, and litter to microbial community assembly, and provide a comprehensive understanding of how tree species mixing shapes microbial communities.

Our study showed that although mixed-species planting did not generally result in significant improvements in soil physicochemical properties or microbial alpha diversity, it significantly altered microbial community composition. Notably, uneven-aged mixed plantations exhibited a more profound influence on microbial communities than even-aged mixed plantations. Consequently, to promote the sustainable development of planted forests, we strongly recommend establishing mixed-species plantations, especially those with uneven-aged tree species. Furthermore, the combination of tree species must be reasonable, and tree species with different mycorrhizal types should be adopted preferentially. Such strategies can optimize microbial community function, enhance soil fertility (assessed by key physicochemical properties, including soil carbon, nitrogen, and phosphorus content), and increase forest productivity (i.e., above-ground biomass increment and growth rates of dominant tree species) and ecological services, ultimately contributing to environmental, economic, and social benefits. This comprehensive approach underscores the importance of tree species diversity in sustainable forest management.

## 5 Conclusion

Contrary to our first hypothesis, we found that microbial alpha diversity was not significantly affected by tree species mixing. However, significant changes were observed in community composition. As predicted by our second hypothesis, the effects of these mixing strategies on the microbial communities varied depending on the microhabitat. In line with our third hypothesis, fungal communities were more sensitive to both types of mixing, whereas bacterial communities showed a stronger correlation with soil environmental conditions. Mantel tests and RDA confirmed that the soil pH was the key driver of changes in microbial communities across all plantations. Given its dominant role in shaping microbial communities and its ease of measurement, we propose that soil pH serve as a highly practical and informative indicator for forest managers. Our findings highlight that the benefits of mixed-species plantations for soil microbes stem more from restructuring communities and enhancing their interactions, rather than merely increasing the local species richness. These findings provide critical mechanistic insights for designing sustainable forest management practices that enhance ecosystem resilience and soil functioning. Future research should endeavor to quantify the relative importance of soil properties, root exudates, and litter in microbial community assembly to offer a comprehensive perspective on the underlying mechanisms by which tree species mixing shapes microbial communities.

## Data Availability

The datasets presented in this study can be found in online repositories. The names of the repository/repositories and accession number(s) can be found below: https://www.ncbi.nlm.nih.gov/, PRJNA1176343 https://www.ncbi.nlm.nih.gov/, PRJNA1176356.
