# Supplementary material for: Effects of even- versus uneven-aged tree species (Pinus massoniana and Castanopsis hystrix) mixing on microbial communities across bulk soil, rhizosphere soil, and fine roots
Source: Front Microbiol. 2025 Dec 3;16:1640866. doi: 10.3389/fmicb.2025.1640866 (PMC12708581; doi:10.3389/fmicb.2025.1640866)
Supplement: Supplementary file 1 [file Data_Sheet_1.docx]

Supplementary Material

# Supplementary Methods

**Methods S1** Detailed steps for the determination of soil extracellular enzymes.

The experimental steps were as follows: First, a soil suspension for enzyme extraction was prepared by homogenizing 1 g of fresh soil in 125 mL of sodium acetate buffer (0.05 M, pH = 5.0). Next, 200 μL of soil suspension and 50 μL of 200 μM fluorometric substrate solution were dispensed into each well of a black polystyrene 96-well microplate using an eight-channel pipettor. The specific fluorometric substrates used for AG, BG, NAG, LAP, and ACP were 4-MUB-α-D-glucopyranoside, 4-MUB-β-D-glucopyranoside, 4-MUB-N-acetyl-β-D-glucosaminide, L-leucine-7-amido-4-methylcoumarin, and 4-MUB-phosphate, respectively. Using the same method, soil controls (200 μL of soil suspension and 50 μL of buffer), substrate controls (200 μL of buffer and 50 μL of substrate solution), and reference standards (200 μL of buffer and 50 μL of standard solution) were prepared, with eight replicates for each. After two hours of incubation in the dark at 25 °C, the reaction was stopped by adding 10 μL of 1 M NaOH to each well. Fluorescence was then measured using a multifunctional microplate reader (Infinite M200 Pro; Tecan, Männedorf, Switzerland) at excitation and emission wavelengths of 365 nm and 450 nm, respectively. Soil extracellular enzyme activities were calculated from the fluorescence values and expressed as nanomoles of the substrate released per gram of dry soil per hour (nmol fluorescence g^-1^ dry soil hr^-1^).

**Methods S2** Detailed methodologies for PCR amplification and high-throughput sequencing.

The PCR reaction was carried out in triplicate, with each 50 μL reaction mixture consisting of 10 μL of 5 × Q5@ reaction buffer, 10 μL of 5 × Q5@ High GC Enhancer, 1.5 μL of 2.5 mM dNTPs, 1.5 μL of each primer (10 μM), 0.2 μL of Q5@ High-Fidelity DNA Polymerase (New England Biolabs, USA), and 50 ng of template DNA, and finally diluted to 50 μL with sterile ddH_2_O. The PCR conditions included an initial denaturation at 95 °C for 5 min, followed by 30 cycles of denaturation at 95 °C for 1 min, annealing at 60 °C for 1 min, extension at 72 °C for 1 min, and a final extension at 72 °C for 7 min. The PCR products were analyzed on a 2% (w/v) agarose gel electrophoresis, purified with the AxyPrep DNA Gel Extraction Kit (Axygen Biosciences, Union City, CA, USA), and quantified using the ABI StepOnePlus Real-Time PCR System (Life Technologies, Foster City, USA). The purified PCR products were pooled at equimolar concentrations and then paired-end sequenced (2 × 250 bp) on an Illumina NovaSeq 6000 platform (Illumina, San Diego, CA, USA) at Gene Denovo Biotechnology Co., Ltd. (Guangzhou, China).

# Supplementary Figures and Tables

## Supplementary Figures


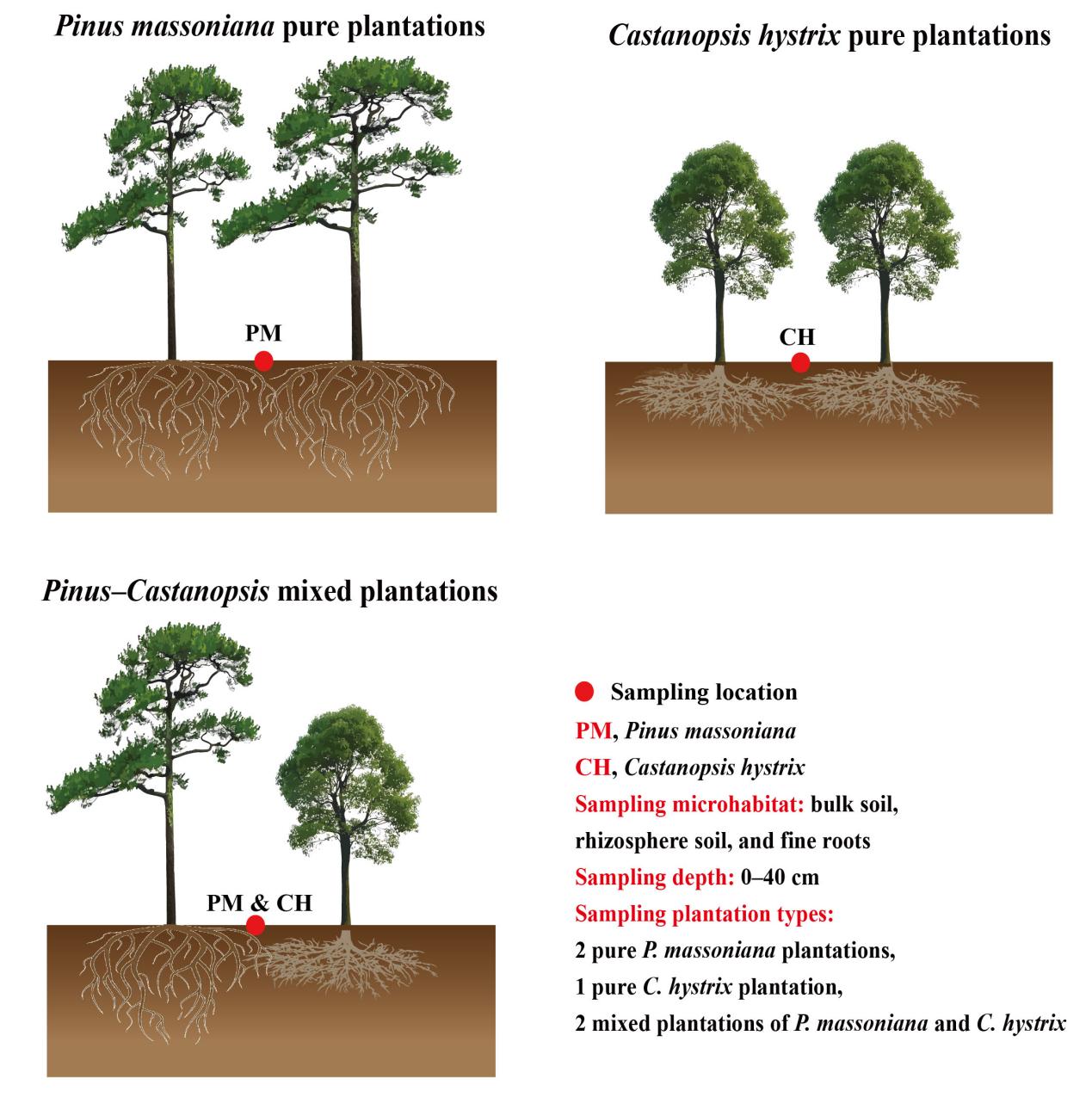


**Supplementary Figure 1.** Schematic diagram of sampling design for this study.

**
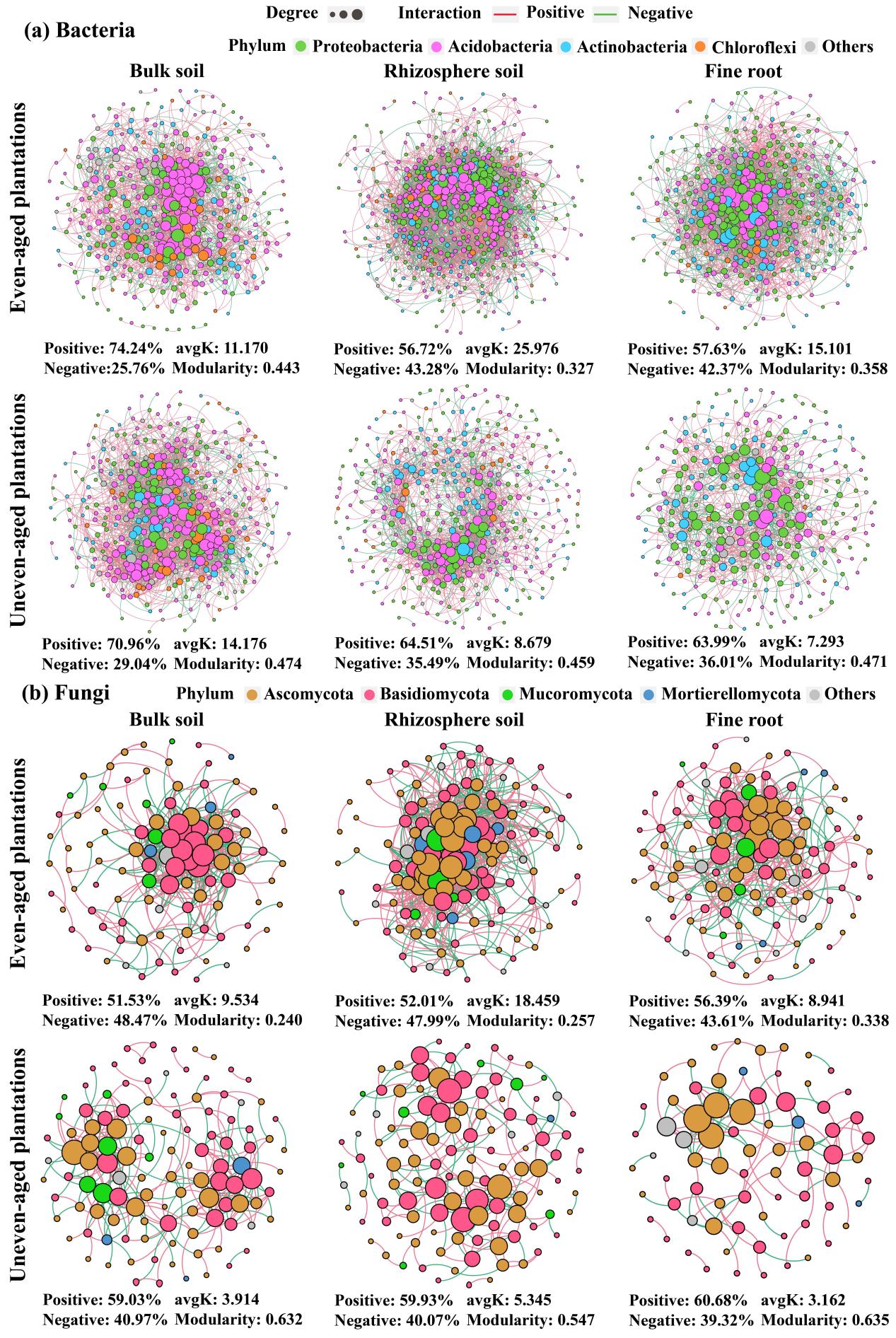
**

**Supplementary Figure 2.** Co-occurrence networks of bacterial (a) and fungal (b) communities in each microhabitat. Nodes indicate individual ASVs, the size of each node is proportional to its degree of connectivity, and the color of the node represents different phylum taxa. Edges represent significant co-occurrence relationships (*p* < 0.05, Spearman's correlation coefficient > 0.6) between ASVs, with red and green edges represent positive and negative relationships, respectively. “avgK” shows the average degree of the network, “Modularity” indicates the degree to which nodes tend to differentiate into different network modules.


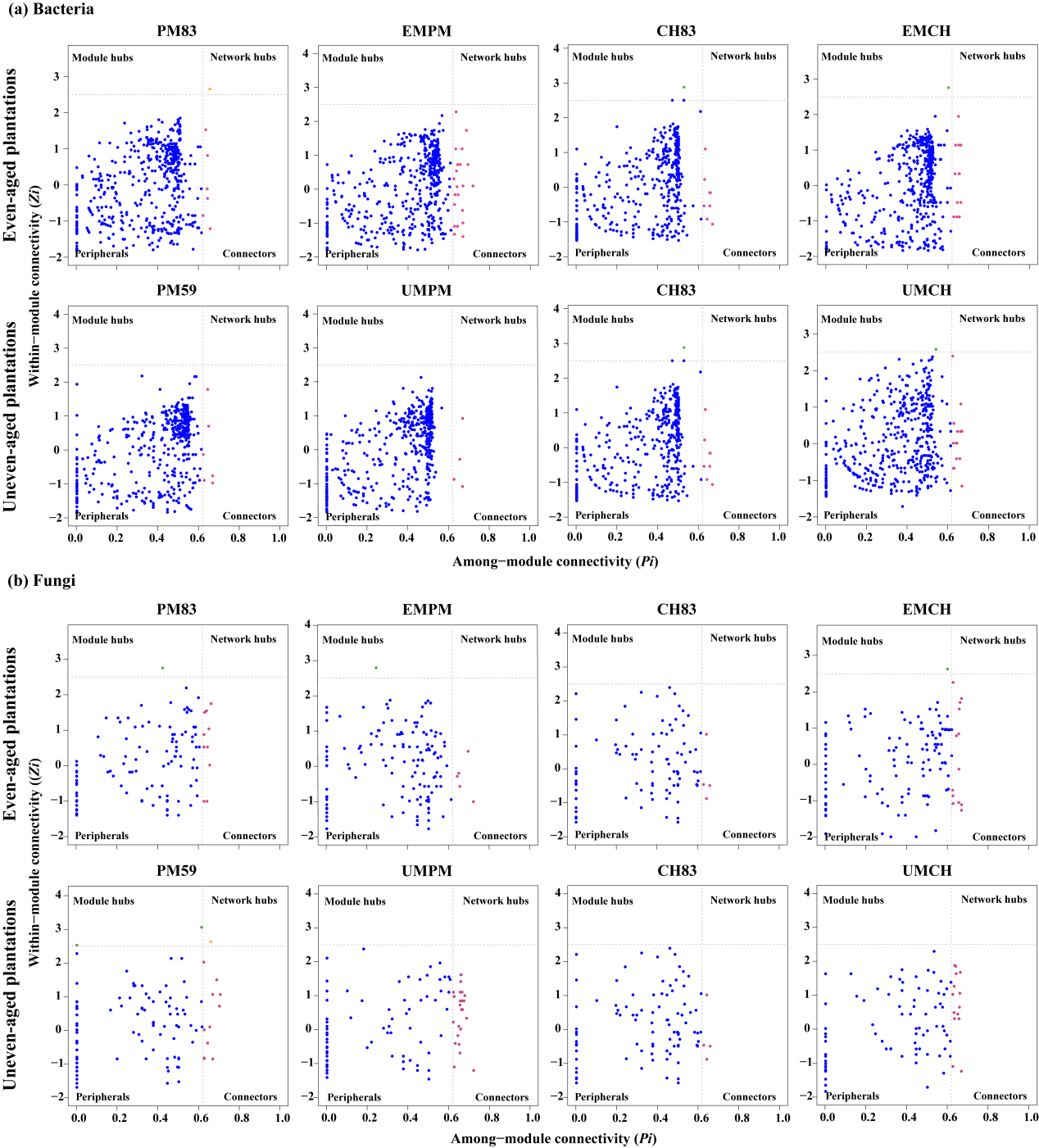


**Supplementary Figure 3.** Within-module (*Zi*) and Among-module connectivity (*Pi*) of the bacterial (a) and fungal (b) co-occurrence networks in each plantation. The *Zi*-*Pi* plot shows the classification of nodes to identify potential keystone taxa within the co-occurrence network. The thresholds of *Zi* and *Pi* for classifying ASVs were 2.5 and 0.62, respectively. Module hubs are shown in green, connectors in pink and network hubs in orange. Abbreviations for each plantation type are consistent with those used in Table S2.


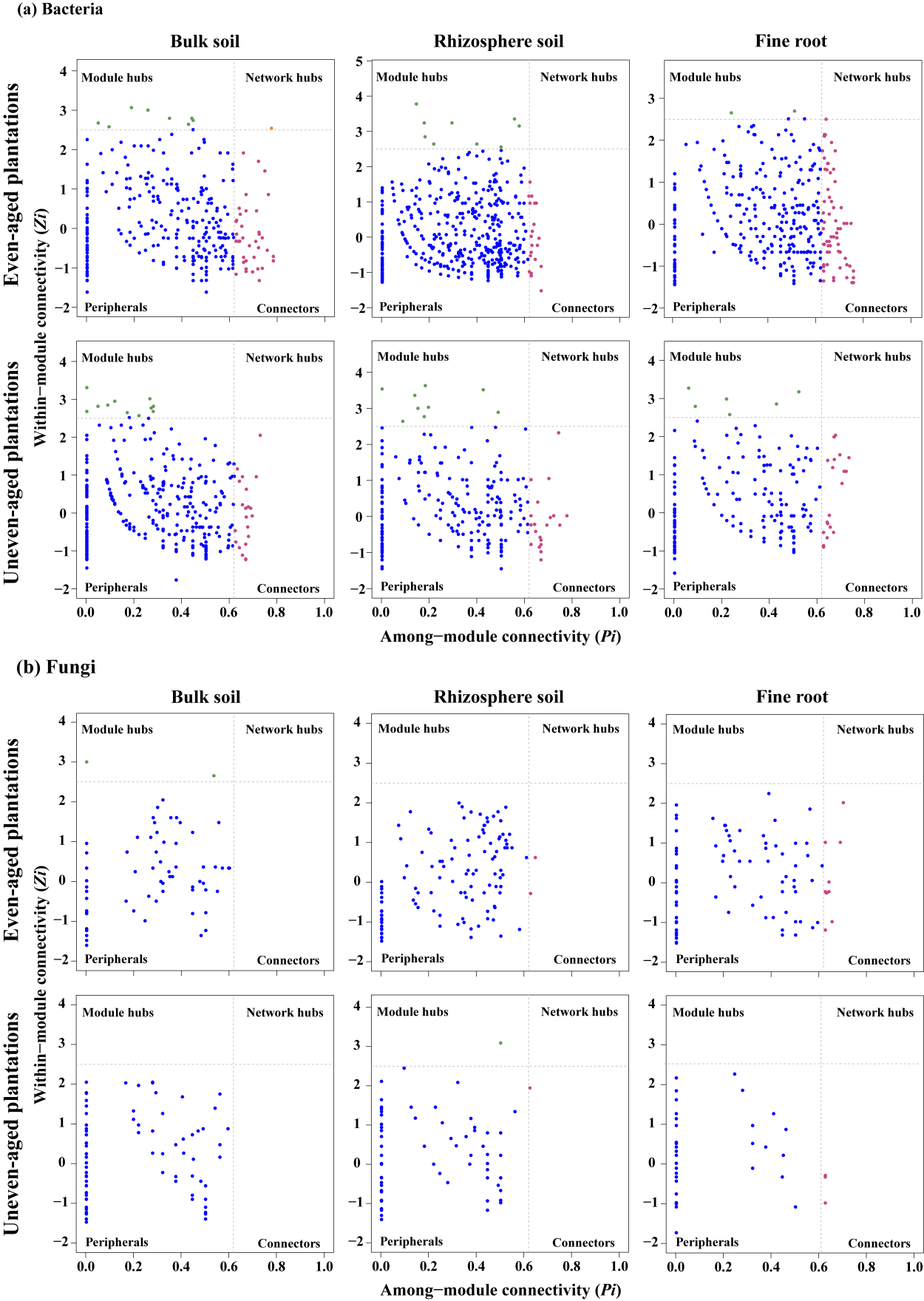


**Supplementary Figure 4.** Within-module (*Zi*) and Among-module connectivity (*Pi*) of the bacterial (a) and fungal (b) co-occurrence networks in each microhabitat. The *Zi*-*Pi* plot shows the classification of nodes to identify potential keystone taxa within the co-occurrence network. The thresholds of *Zi* and *Pi* for classifying ASVs were 2.5 and 0.62, respectively. Module hubs are shown in green, connectors in pink, and network hubs in orange.

## Supplementary Tables

**Table S1** Stand characteristics of the five plantation types in the study.

| Plantation type | Geographic location | Altitude (m) | Slope (°) | Canopy  coverage (%) | Density (tree ha^-1^) | Mean DBH (cm) | Mean height (m) | Basal area (m^2^ ha^-1^) |
| --- | --- | --- | --- | --- | --- | --- | --- | --- |
| PM83 | 106°51'20" E, 22°3'5" N | 460–472 | 28.3–33.6 | 75.24 ± 0.06 | 617 ± 15 | 33.1 ± 5.3 | 21.2 ± 2.4 | 54.4 ± 3.2 |
| PM59 | 106°51'47" E, 22°2'43" N | 515–530 | 28.4–29.6 | 77.75 ± 0.04 | 358 ± 38 | 45.4 ± 9.0 | 25.6 ± 3.2 | 60.1 ± 9.0 |
| CH83 | 106°44'34" E, 22°4'47" N | 472–560 | 28.5–30.3 | 83.31 ± 0.03 | 409 ± 30 | 28.5 ± 9.9 | 19.7 ± 4.5 | 28.8 ± 6.8 |
| EMP | 106°51'48" E, 22°2'43" N | 537–539 | 24.6–26.3 | 83.79 ± 0.03 | 175 ± 1^pm^ | 28.8 ± 5.5^pm^ | 19.5 ± 2.9^pm^ | 11.8 ± 0.7^pm^ |
|  |  |  |  |  | 258 ± 15^ch^ | 32.4 ± 9.0^ch^ | 20.6 ± 3.2^ch^ | 23.2 ± 2.4^ch^ |
| UMP | 106°51'31" E, 22°2'44" N | 441–503 | 31.2–32.5 | 81.41 ± 0.04 | 242 ± 29^pm^ | 41.1 ± 5.7^pm^ | 20.6 ± 3.3^pm^ | 33.8 ± 3.9^pm^ |
|  |  |  |  |  | 325 ± 25^ch^ | 22.1 ± 6.5^ch^ | 18.1 ± 2.2^ch^ | 13.5 ± 0.5^ch^ |

Note: Values are expressed as mean ± standard deviation (n = 3). DBH: Diameter at breast height (1.3 m above ground). PM83, pure *P. massoniana* plantation planted in 1983; CH83, pure *C. hystrix* plantation planted in 1983; EMP, even-aged mixed *P. massoniana* and *C. hystrix* plantation; PM59, pure *P. massoniana* plantation planted in 1959; UMP, uneven-aged mixed *P. massoniana* and *C. hystrix* plantation; pm: *P. massoniana* in mixed plantations; ch: *C. hystrix* in mixed plantations.

**Table S2** Chemical properties and enzyme activities of bulk and rhizosphere soils in two mixed plantations and their respective monocultures.

| Microhabitat | Plantation type | pH | SOM  (g.kg^-1^) | TN  (g.kg^-1^) | TP  (g.kg^-1^) | NH_4_^+^-N (mg.kg^-1^) | NO_3_^-^-N  (mg.kg^-1^) | AN  (mg.kg^-1^) | AP  (mg.kg^-1^) | AG (nmol g^-1^ h^-1^) | BG  (nmol g^-1^ h^-1^) | NAG (nmol g^-1^ h^-1^) | LAP (nmol g^-1^ h^-1^) | ACP (nmol g^-1^ h^-1^) |
| --- | --- | --- | --- | --- | --- | --- | --- | --- | --- | --- | --- | --- | --- | --- |
| **Even-aged plantations** | | | | | | | | | | | | | | |
| Bulk soil | PM83 | 3.58 ± 0.02 Ab | 32.70 ± 4.79 Ba | 1.22 ± 0.14 Ba | 0.21 ± 0.01 Ab | 8.24 ± 0.46 Ba | 1.95 ± 0.38 Aab | 10.19 ± 0.47 Ba | 1.86 ± 0.14 Aa | 27.63 ± 2.56 Ba | 757.36 ± 73.02 Ba | 481.12 ± 41.98 Ba | 145.36 ± 3.88 Ab | 9963.78 ± 583.72 Aa |
|  | EMPM | 3.64 ± 0.03 Aab | 36.61 ± 5.88 Ba | 1.47 ± 0.18 Ba | 0.27 ± 0.01 Ba | 10.44 ± 1.35 Ba | 2.57 ± 0.35 Aa | 13.01 ± 1.66 Ba | 1.82 ± 0.12 Ba | 25.53 ± 2.20 Ba | 855.05 ± 89.97 Ba | 540.89 ± 88.73 Ba | 155.56 ± 5.68 Ab | 9416.76 ± 259.15 Ba |
|  | CH83 | 3.68 ± 0.03 Aa | 29.92 ± 4.92 Ba | 1.29 ± 0.16 Ba | 0.23 ± 0.02 Aab | 8.55 ± 0.55 Ba | 1.16 ± 0.06 Ab | 9.71 ± 0.56 Ba | 1.76 ± 0.13 Aa | 27.30 ± 3.85 Ba | 644.21 ± 79.05 Ba | 615.04 ± 112.23 Ba | 271.16 ± 3.31 Aa | 7565.16 ± 342.99 Bb |
|  | EMCH | 3.64 ± 0.03 Aab | 36.61 ± 5.88 Ba | 1.47 ± 0.18 Ba | 0.27 ± 0.01 Ba | 10.44 ± 1.35 Ba | 2.57 ± 0.35 Aa | 13.01 ± 1.66 Aa | 1.82 ± 0.12 Ba | 25.53 ± 2.20 Ba | 855.05 ± 89.97 Ba | 540.89 ± 88.73 Ba | 155.56 ± 5.68 Ab | 9416.76 ± 259.15 Aa |
| Rhizosphere soil | PM83 | 3.30 ± 0.07 Bab | 52.61 ± 5.74 Ab | 1.76 ± 0.19 Ab | 0.21 ± 0.01 Ac | 16.92 ± 1.43 Ab | 1.31 ± 0.22 Aa | 18.23 ± 1.63 Ab | 2.15 ± 0.26 Abc | 109.49 ± 17.50 Aa | 2553.86 ± 341.15 Aa | 1615.31 ± 438.76 Ab | 156.28 ± 5.82 Aa | 11531.44 ± 484.88 Aa |
|  | EMPM | 3.25 ± 0.07 Bb | 84.60 ± 6.67 Aa | 3.37 ± 0.42 Aa | 0.34 ± 0.02 Aa | 27.84 ± 3.19 Aab | 1.11 ± 0.31 Ba | 28.95 ± 3.46 Aab | 2.99 ± 0.53 Aab | 99.34 ± 1.84 Aa | 2857.32 ± 294.78 Aa | 2721.59 ± 206.94 Aa | 159.97 ± 4.19 Aa | 10937.46 ± 305.50 Aab |
|  | CH83 | 3.40 ± 0.02 Ba | 56.53 ± 3.30 Ab | 2.29 ± 0.20 Ab | 0.27 ± 0.02 Ab | 30.31 ± 1.53 Aa | 1.07 ± 0.06 Aa | 31.38 ± 1.55 Aa | 2.11 ± 0.16 Ac | 88.52 ± 10.29 Aa | 1600.05 ± 290.69 Ab | 988.43 ± 57.47 Ab | 169.66 ± 2.06 Ba | 10884.46 ± 408.73 Aab |
|  | EMCH | 3.07 ± 0.03 Bc | 94.95 ± 0.74 Aa | 3.71 ± 0.07 Aa | 0.35 ± 0.01 Aa | 28.76 ± 9.95 Aab | 1.57 ± 0.81 Aa | 30.34 ± 10.76 Aab | 3.44 ± 0.28 Aa | 79.07 ± 6.68 Aa | 2206.06 ± 122.89 Aab | 1501.01 ± 450.68 Ab | 183.72 ± 29.24 Aa | 9622.41 ± 434.22 Ab |
| **Uneven-aged plantations** | | | | | | | | | | | | | | |
| Bulk soil | PM59 | 3.53 ± 0.03 Ab | 25.35 ± 3.67 Ba | 1.04 ± 0.10 Ba | 0.21 ± 0.01 Ba | 6.62 ± 0.54 Bb | 1.59 ± 0.10 Ba | 8.21 ± 0.53 Bb | 1.94 ± 0.14 Aa | 21.38 ± 2.97 Ba | 575.23 ± 85.45 Bb | 423.44 ± 65.05 Ba | 249.99 ± 3.64 Ab | 6864.38 ± 370.28 Ba |
|  | UMPM | 3.51 ± 0.03 Ab | 27.62 ± 2.50 Ba | 1.17 ± 0.08 Ba | 0.23 ± 0.01 Aa | 6.85 ± 0.33 Bb | 1.71 ± 0.26 Ba | 8.55 ± 0.24 Bab | 2.04 ± 0.13 Aa | 23.46 ± 4.09 Ba | 925.87 ± 102.60 Ba | 834.20 ± 248.71 Aa | 130.42 ± 2.16 Ac | 7908.34 ± 746.46 Aa |
|  | CH83 | 3.68 ± 0.03 Aa | 29.92 ± 4.92 Ba | 1.29 ± 0.16 Ba | 0.23 ± 0.02 Aa | 8.55 ± 0.55 Ba | 1.16 ± 0.06 Aa | 9.71 ± 0.56 Ba | 1.76 ± 0.13 Aa | 27.30 ± 3.85 Ba | 644.21 ± 79.05 Bb | 615.04 ± 112.23 Ba | 271.16 ± 3.31 Aa | 7565.16 ± 342.99 Ba |
|  | UMCH | 3.51 ± 0.03 Ab | 27.62 ± 2.50 Ba | 1.17 ± 0.08 Ba | 0.23 ± 0.01 Ba | 6.85 ± 0.33 Bb | 1.71 ± 0.26 Ba | 8.55 ± 0.24 Bab | 2.04 ± 0.13 Aa | 23.46 ± 4.09 Ba | 925.87 ± 102.60 Ba | 834.20 ± 248.71 Aa | 130.42 ± 2.16 Ac | 7908.34 ± 746.46 Aa |
| Rhizosphere soil | PM59 | 3.34 ± 0.02 Ba | 52.55 ± 1.67 Aa | 1.68 ± 0.10 Ab | 0.25 ± 0.01 Aa | 27.18 ± 0.22 Aa | 2.83 ± 0.32 Ab | 30.01 ± 0.40 Aab | 2.26 ± 0.05 Aa | 88.91 ± 2.62 Aa | 3150.96 ± 37.87 Aa | 1917.05 ± 84.24 Aa | 135.60 ± 3.16 Bb | 10009.39 ± 276.74 Aab |
|  | UMPM | 3.16 ± 0.03 Bb | 52.67 ± 4.19 Aa | 1.75 ± 0.13 Aab | 0.26 ± 0.01 Aa | 23.46 ± 1.02 Ab | 3.15 ± 0.12 Ab | 26.62 ± 1.00 Ab | 1.79 ± 0.04 Ab | 71.90 ± 4.98 Aab | 1961.58 ± 234.36 Ab | 1392.84 ± 167.94 Ab | 130.72 ± 5.72 Ab | 8796.34 ± 802.45 Abc |
|  | CH83 | 3.40 ± 0.02 Ba | 56.53 ± 3.30 Aa | 2.29 ± 0.20 Aa | 0.27 ± 0.02 Aa | 30.31 ± 1.53 Aa | 1.07 ± 0.06 Ac | 31.38 ± 1.55 Aa | 2.11 ± 0.16 Aab | 88.52 ± 10.29 Aa | 1600.05 ± 290.69 Ab | 988.43 ± 57.47 Ac | 169.66 ± 2.06 Ba | 10884.46 ± 408.73 Aa |
|  | UMCH | 3.17 ± 0.05 Bb | 63.30 ± 7.27 Aa | 2.23 ± 0.29 Aab | 0.27 ± 0.01 Aa | 18.87 ± 1.44 Ac | 3.73 ± 0.14 Aa | 22.60 ± 1.50 Ac | 1.92 ± 0.19 Aab | 63.64 ± 6.60 Ab | 1814.39 ± 173.51 Ab | 1167.15 ± 155.15 Abc | 122.64 ± 8.09 Ab | 8139.43 ± 781.26 Ac |

Note: Values are expressed as mean ± standard error (*n* = 6). Different lowercase letters within a column denote significant differences among various plantation types within the same microhabitat. Distinct uppercase letters within a column show significant differences between bulk and rhizosphere soils within the same plantation type. Statistical analysis was performed using one-way ANOVA (*p* < 0.05), followed by Tukey’s post-hoc test for further comparison. Abbreviations: PM83, pure *P. massoniana* plantation established in 1983; EMPM, *P. massoniana* in the even-aged mixed plantation consisting of *P. massoniana* and *C. hystrix*; CH83, pure *C. hystrix* plantation planted in 1983; EMCH, *C. hystrix* in the even-aged mixed plantation consisting of *P. massoniana* and *C. hystrix*; PM59, pure *P. massoniana* plantation planted in 1959; UMPM, *P. massoniana* in the uneven-aged mixed plantation consisting of *P. massoniana* and *C. hystrix*; UMCH, *C. hystrix* in the uneven-aged mixed plantation consisting of *P. massoniana* and *C. hystrix*. pH, soil pH; SOM, soil organic matter; TN, total nitrogen; TP, total phosphorus; NH_4_^+^-N, ammonia nitrogen; NO_3_^-^-N, nitrate nitrogen; AN, available nitrogen; AP, available phosphorus; AG, α-1,4-glucosidase; BG, β-1,4-glucosidase; NAG, β-1,4-N-acetyl-glucosaminidase; LAP, leucine aminopeptidase; ACP, acid phosphatase.

**Table S3** The results of the linear mixed-effects model show the effects of plantation type, microhabitat, and their interactions on soil chemical properties and enzyme activities.

|  | | pH | SOM | TN | TP | NH_4_^+^-N | NO_3_^-^-N | AN | AP | AG | BG | NAG | LAP | ACP |
| --- | --- | --- | --- | --- | --- | --- | --- | --- | --- | --- | --- | --- | --- | --- |
| **Even-aged plantations** | | | | | | | | | | | | | | |
| Plantation type | *F* | 7.01 | 2.31 | 4.91 | 2.35 | 1.69 | 0.34 | 1.50 | 3.64 | 0.70 | 3.09 | 2.08 | 3.14 | 2.08 |
|  | *p* | **<0.001** | 0.095 | **<0.01** | 0.091 | 0.188 | 0.796 | 0.233 | **<0.05** | 0.558 | **<0.05** | 0.123 | **<0.05** | 0.122 |
| Microhabitat | *F* | 211.55 | 52.97 | 65.38 | 15.30 | 98.41 | 8.65 | 70.87 | 37.91 | 208.50 | 94.00 | 71.64 | 3.43 | 27.88 |
|  | *p* | **<0.001** | **<0.001** | **<0.001** | **<0.001** | **<0.001** | **<0.01** | **<0.001** | **<0.001** | **<0.001** | **<0.001** | **<0.001** | 0.073 | **<0.001** |
| Plantation type × Microhabitat | *F* | 7.51 | 1.07 | 2.22 | 1.69 | 1.66 | 1.83 | 2.09 | 5.32 | 0.43 | 0.82 | 3.29 | 26.12 | 5.31 |
|  | *p* | **<0.001** | 0.374 | 0.105 | 0.189 | 0.195 | 0.161 | 0.122 | **<0.01** | 0.732 | 0.492 | **<0.05** | **<0.001** | **<0.01** |
| **Uneven-aged plantations** | | | | | | | | | | | | | | |
| Plantation type | *F* | 3.41 | 0.56 | 1.81 | 0.57 | 8.75 | 4.09 | 5.90 | 0.49 | 1.67 | 1.71 | 0.53 | 5.12 | 17.86 |
|  | *p* | **<0.05** | 0.691 | 0.161 | 0.687 | **<0.001** | **<0.05** | **<0.01** | 0.692 | 0.188 | 0.180 | 0.704 | **<0.01** | **<0.001** |
| Microhabitat | *F* | 158.79 | 92.63 | 65.49 | 14.78 | 933.12 | 54.62 | 1173.39 | 0.70 | 198.37 | 110.79 | 39.71 | 180.30 | 17.86 |
|  | *p* | **<0.001** | **<0.001** | **<0.001** | **<0.001** | **<0.001** | **<0.001** | **<0.001** | 0.407 | **<0.001** | **<0.001** | **<0.001** | **<0.001** | **<0.001** |
| Plantation type × Microhabitat | *F* | 2.95 | 0.24 | 0.58 | 0.32 | 4.69 | 8.78 | 4.51 | 2.96 | 2.56 | 6.92 | 3.51 | 48.20 | 2.90 |
|  | *p* | **<0.05** | 0.866 | 0.630 | 0.812 | **<0.01** | **<0.001** | **<0.01** | **<0.05** | 0.069 | **<0.001** | **<0.05** | **<0.001** | **<0.05** |
|  |  |  |  |  |  |  |  |  |  |  |  |  |  |  |

Note: *F*, variance ratio; *P*, probability of type-III error (two-sided); ×, denotes an interaction term. The same as below.

**Table S4** Alpha diversity of the bacterial and fungal communities inhabiting the three microhabitats in different plantations.

| Microhabitat | Plantation type | Bacteria | | Fungi | |
| --- | --- | --- | --- | --- | --- |
|  |  | Chao1 | Shannon | Chao1 | Shannon |
| **Even-aged plantations** | | | | | |
| Bulk soil | PM83 | 1353.09 ± 24.29 A | 8.94 ± 0.07 A | 375.63 ± 14.41 A | 5.08 ± 0.21 A |
|  | EMPM | 1306.48 ± 46.15 A | 8.78 ± 0.10 B | 394.11 ± 13.02 A | 4.83 ± 0.35 AB |
|  | CH83 | 1299.59 ± 14.80 A | 8.77 ± 0.02 B | 379.70 ± 7.15 A | 4.44 ± 0.16 A |
|  | EMCH | 1306.48 ± 46.15 A | 8.78 ± 0.10 B | 394.11 ± 13.02 A | 4.83 ± 0.35 A |
| Rhizosphere soil | PM83 | 1364.20 ± 70.74 A | 9.04 ± 0.09 A | 318.06 ± 15.80 A | 4.06 ± 0.41 A |
|  | EMPM | 1387.85 ± 26.45 A | 9.16 ± 0.08 A | 331.04 ± 5.16 B | 4.12 ± 0.45 B |
|  | CH83 | 1323.63 ± 17.53 A | 9.15 ± 0.07 A | 340.49 ± 10.03 AB | 4.64 ± 0.21 A |
|  | EMCH | 1422.25 ± 37.05 A | 9.51 ± 0.04 A | 387.54 ± 14.81 A | 4.22 ± 0.27 A |
| Fine root | PM83 | 1177.02 ± 41.85 B | 8.54 ± 0.09 B | 312.65 ± 21.25 A | 4.88 ± 0.22 A |
|  | EMPM | 1238.60 ± 21.76 A | 8.65 ± 0.15 AB | 337.66 ± 15.87 AB | 5.79 ± 0.17 A |
|  | CH83 | 1321.54 ± 52.84 A | 8.85 ± 0.10 AB | 255.90 ± 22.70 B | 4.78 ± 0.23 A |
|  | EMCH | 1378.81 ± 49.53 A | 8.95 ± 0.10 AB | 330.58 ± 11.91 A | 4.23 ± 0.46 A |
| **Uneven-aged plantations** | | | | | |
| Bulk soil | PM59 | 1369.92 ± 15.90 A | 8.88 ± 0.09 A | 425.09 ± 6.75 A | 5.41 ± 0.16 A |
|  | UMPM | 1432.85 ± 17.30 A | 9.03 ± 0.06 A | 369.36 ± 12.02 A | 5.37 ± 0.23 A |
|  | CH83 | 1299.59 ± 14.80 A | 8.77 ± 0.02 B | 379.70 ± 7.15 A | 4.44 ± 0.16 A |
|  | UMCH | 1432.85 ± 17.30 A | 9.03 ± 0.06 A | 369.36 ± 12.02 A | 5.37 ± 0.23 A |
| Rhizosphere soil | PM59 | 1336.74 ± 63.12 A | 9.11 ± 0.07 A | 353.04 ± 30.76 AB | 4.71 ± 0.24 A |
|  | UMPM | 1329.12 ± 29.26 AB | 9.15 ± 0.12 A | 309.80 ± 13.66 AB | 4.97 ± 0.18 A |
|  | CH83 | 1323.63 ± 17.53 A | 9.15 ± 0.07 A | 340.49 ± 10.03 AB | 4.64 ± 0.21 A |
|  | UMCH | 1311.70 ± 25.51 B | 9.17 ± 0.11 A | 314.04 ± 18.19 AB | 4.43 ± 0.23 AB |
| Fine root | PM59 | 1264.28 ± 26.02 A | 9.08 ± 0.17 A | 328.52 ± 9.65 B | 5.29 ± 0.20 A |
|  | UMPM | 1201.33 ± 53.53 B | 8.74 ± 0.08 B | 270.9 ± 5.70 B | 4.67 ± 0.15 A |
|  | CH83 | 1321.54 ± 52.84 A | 8.85 ± 0.10 A | 255.90 ± 22.70 B | 4.78 ± 0.23 A |
|  | UMCH | 1277.03 ± 59.49 B | 8.82 ± 0.08 A | 248.49 ± 21.12 B | 4.29 ± 0.32 B |

Note: Values are expressed as mean ± standard error (n = 6). Different uppercase letters within a column indicate significant differences among different microhabitats within the same plantation. Statistical significance was determined by the non-parametric Kruskal-Wallis test (*p* < 0.05). Abbreviations for each plantation type are the same as those used in Table S2.

**Table S5** The results of the linear mixed-effects model show the effects of plantation type, microhabitat, and their interactions on alpha diversity of the bacterial and fungal communities.

|  | | Bacteria | | Fungi | |
| --- | --- | --- | --- | --- | --- |
|  |  | Chao1 | Shannon | Chao1 | Shannon |
| **Even-aged plantations** | | | | | |
| Plantation type | *F* | 1.00 | 2.26 | 1.44 | 0.78 |
|  | *p* | 0.403 | 0.095 | 0.243 | 0.513 |
| Microhabitat | *F* | 4.25 | 26.49 | 25.05 | 4.66 |
|  | *p* | **<0.05** | **<0.001** | **<0.001** | **<0.05** |
| Plantation type × Microhabitat | *F* | 2.09 | 3.08 | 2.37 | 2.13 |
|  | *p* | 0.07 | **<0.05** | **<0.05** | 0.069 |
| **Uneven-aged plantations** | | | | | |
| Plantation type | *F* | 0.20 | 0.44 | 3.46 | 1.24 |
|  | *p* | 0.897 | 1.000 | 1.000 | 0.303 |
| Microhabitat | *F* | 10.42 | 9.66 | 38.85 | 4.78 |
|  | *p* | **<0.001** | **<0.001** | **<0.001** | **<0.05** |
| Plantation type × Microhabitat | *F* | 2.26 | 1.84 | 1.12 | 2.65 |
|  | *p* | 0.050 | 0.107 | 0.364 | **<0.05** |

**Table S6** The relative abundance (%) at the phylum level of bacterial communities inhabiting the three microhabitats in different plantations.

| Microhabitat | Plantation type | Acidobacteria | Proteobacteria | Actinobacteria | Chloroflexi | Firmicutes | Verrucomicrobia | Dependentiae | Armatimonadetes | Gemmatimonadetes | Bacteroidetes | Others | Unclassified |
| --- | --- | --- | --- | --- | --- | --- | --- | --- | --- | --- | --- | --- | --- |
| **Even-aged plantations** | | | | | | | | | | | | | |
| Bulk soil | PM83 | 40.30 ± 1.76 Aa | 16.73 ± 1.17 Ba | 20.83 ± 1.91 Aa | 10.18 ± 1.38 Aa | 5.89 ± 0.84 Aa | 0.53 ± 0.04 ABa | 0.30 ± 0.09 ABa | 0.05 ± 0.01 Ba | 0.05 ± 0.01 Ba | 0.04 ± 0.01 Aa | 0.06 ± 0.01 Aab | 5.03 ± 0.38 Aa |
|  | EMPM | 38.20 ± 3.06 ABa | 16.17 ± 1.76 Ba | 18.80 ± 2.67 Aa | 7.26 ± 0.86 Aa | 13.27 ± 4.98 Aa | 0.60 ± 0.08 Aa | 0.48 ± 0.06 ABa | 0.05 ± 0.01 Ba | 0.03 ± 0.00 Ba | 0.03 ± 0.01 ABa | 0.03 ± 0.01 Bb | 5.09 ± 0.46 Aa |
|  | CH83 | 45.84 ± 3.54 Aa | 18.78 ± 1.69 ABa | 14.38 ± 0.97 Aa | 6.05 ± 0.87 Aa | 9.20 ± 3.16 Aa | 0.49 ± 0.03 Ba | 0.30 ± 0.05 Aa | 0.02 ± 0.00 Aa | 0.04 ± 0.01 Ba | 0.02 ± 0.00 Aa | 0.10 ± 0.02 Aa | 4.78 ± 0.55 Aa |
|  | EMCH | 38.20 ± 3.06 ABa | 16.17 ± 1.76 Ba | 18.80 ± 2.67 Ba | 7.26 ± 0.86 Aa | 13.27 ± 4.98 Aa | 0.60 ± 0.08 Ba | 0.48 ± 0.06 Aa | 0.05 ± 0.01 Ba | 0.03 ± 0.00 Ba | 0.03 ± 0.01 Aa | 0.03 ± 0.01 Bb | 5.09 ± 0.46 Aa |
| Rhizosphere soil | PM83 | 45.37 ± 2.59 Aa | 36.87 ± 3.02 Aa | 8.83 ± 1.64 Ba | 3.17 ± 1.15 Ba | 0.09 ± 0.02 Bb | 0.84 ± 0.16 Aa | 0.41 ± 0.08 Aa | 0.20 ± 0.03 ABab | 0.06 ± 0.01 Aa | 0.05 ± 0.01 Aab | 0.07 ± 0.02 Aa | 4.04 ± 0.35 ABa |
|  | EMPM | 47.57 ± 2.53 Aa | 30.98 ± 1.40 ABa | 12.79 ± 2.87 Aa | 1.97 ± 0.78 Ba | 0.18 ± 0.04 ABab | 1.28 ± 0.22 Aa | 0.74 ± 0.20 Aa | 0.13 ± 0.01 ABb | 0.05 ± 0.01 ABa | 0.10 ± 0.02 Aa | 0.19 ± 0.06 Aa | 4.02 ± 0.29 ABa |
|  | CH83 | 43.31 ± 2.31 Aa | 29.85 ± 1.54 Aa | 12.21 ± 1.21 Aa | 4.28 ± 1.26 ABa | 3.79 ± 1.62 Aa | 1.07 ± 0.15 Aa | 0.74 ± 0.09 Aa | 0.07 ± 0.02 ABb | 0.06 ± 0.01 ABa | 0.02 ± 0.01 Ab | 0.06 ± 0.01 Aa | 4.55 ± 0.32 Aa |
|  | EMCH | 40.91 ± 2.00 Aa | 35.94 ± 2.35 ABa | 13.38 ± 2.27 ABa | 1.84 ± 0.18 ABa | 0.16 ± 0.01 ABab | 1.83 ± 0.29 Aa | 0.57 ± 0.04 Aa | 0.21 ± 0.01 ABa | 0.06 ± 0.02 Aa | 0.08 ± 0.02 Aab | 0.12 ± 0.02 Ba | 4.90 ± 0.67 ABa |
| Fine root | PM83 | 19.14 ± 1.70 Bab | 56.08 ± 1.46 Aa | 19.44 ± 1.18 Aa | 1.94 ± 0.48 Ba | 0.18 ± 0.06 Ba | 0.24 ± 0.05 Bb | 0.10 ± 0.01 Ca | 0.39 ± 0.06 Aa | 0.25 ± 0.06 Aa | 0.02 ± 0.01 Aa | 0.07 ± 0.02 Aa | 2.17 ± 0.28 Ba |
|  | EMPM | 22.35 ± 1.50 Bab | 60.81 ± 2.34 Aa | 11.12 ± 1.79 Aa | 1.73 ± 0.95 Ba | 0.09 ± 0.01 Ba | 0.44 ± 0.07 Aab | 0.14 ± 0.03 Ba | 0.24 ± 0.08 Aa | 0.19 ± 0.01 Aab | 0.01 ± 0.01 Ba | 0.12 ± 0.03 ABa | 2.76 ± 0.62 Ba |
|  | CH83 | 26.04 ± 0.97 Ba | 50.28 ± 3.58 Aa | 17.48 ± 3.61 Aa | 2.28 ± 0.90 Ba | 0.15 ± 0.08 Ba | 0.76 ± 0.05 Ba | 0.23 ± 0.06 Ba | 0.29 ± 0.05 Ba | 0.11 ± 0.02 Aab | 0.06 ± 0.03 Aa | 0.09 ± 0.02 Aa | 2.22 ± 0.20 Ba |
|  | EMCH | 18.48 ± 1.08 Bb | 52.60 ± 3.97 Aa | 24.46 ± 5.43 Aa | 0.84 ± 0.07 Ba | 0.07 ± 0.04 Ba | 0.65 ± 0.06 ABab | 0.23 ± 0.11 Aa | 0.44 ± 0.02 Aa | 0.06 ± 0.00 ABb | 0.05 ± 0.03 Aa | 0.15 ± 0.02 A | 1.97 ± 0.25 Ba |
| **Uneven-aged plantations** | | | | | | | | | | | | | |
| Bulk soil | PM59 | 46.64 ± 2.70 Aa | 18.31 ± 1.05 Ba | 17.53 ± 2.01 Aa | 6.69 ± 1.02 Aa | 4.85 ± 1.29 Aa | 0.53 ± 0.05 Aa | 0.89 ± 0.15 Aa | 0.07 ± 0.01 Ba | 0.05 ± 0.00 Ba | 0.03 ± 0.01 Aa | 0.04 ± 0.01 Ab | 4.37 ± 0.39 Aa |
|  | UMPM | 45.11 ± 3.74 Aa | 17.87 ± 1.07 Ba | 17.43 ± 2.81 Aa | 8.96 ± 1.88 Aa | 4.35 ± 0.81 Aa | 0.43 ± 0.03 Aa | 1.48 ± 0.56 Aa | 0.06 ± 0.00 Ba | 0.06 ± 0.01 Aa | 0.03 ± 0.01 Ba | 0.08 ± 0.01 Aab | 4.14 ± 0.54 ABa |
|  | CH83 | 45.84 ± 3.54 Aa | 18.78 ± 1.69 Ba | 14.38 ± 0.97 Aa | 6.05 ± 0.87 Aa | 9.20 ± 3.16 Aa | 0.49 ± 0.03 Ba | 0.30 ± 0.05 Aa | 0.02 ± 0.00 Bb | 0.04 ± 0.01 Ba | 0.02 ± 0.00 Aa | 0.10 ± 0.02 Aa | 4.78 ± 0.55 Aa |
|  | UMCH | 45.11 ± 3.74 Aa | 17.87 ± 1.07 Ba | 17.43 ± 2.81 Aa | 8.96 ± 1.88 Aa | 4.35 ± 0.81 Aa | 0.43 ± 0.03 Aa | 1.48 ± 0.56 Aa | 0.06 ± 0.00 Bb | 0.06 ± 0.01 Aa | 0.03 ± 0.01 Aa | 0.08 ± 0.01 Aab | 4.14 ± 0.54 Aa |
| Rhizosphere soil | PM59 | 44.59 ± 3.28 Aa | 34.57 ± 1.64 ABa | 11.01 ± 2.14 Ac | 2.45 ± 0.64 Ba | 0.44 ± 0.23 Bb | 1.07 ± 0.11 Aa | 1.36 ± 0.29 Aa | 0.18 ± 0.04 ABa | 0.10 ± 0.02 ABa | 0.04 ± 0.01 Aa | 0.07 ± 0.01 Aa | 4.12 ± 0.31 Ab |
|  | UMPM | 34.84 ± 2.10 Aa | 29.99 ± 1.58 ABab | 23.91 ± 2.12 Aa | 3.61 ± 0.91 ABa | 0.73 ± 0.12 ABbc | 0.35 ± 0.06 Ab | 0.84 ± 0.18 Aa | 0.11 ± 0.03 Aa | 0.12 ± 0.04 Aa | 0.03 ± 0.01 Ba | 0.13 ± 0.04 Aa | 5.34 ± 0.73 Aab |
|  | CH83 | 43.31 ± 2.31 Aa | 29.85 ± 1.54 ABab | 12.21 ± 1.21 Abc | 4.28 ± 1.26 ABa | 3.79 ± 1.62 Aa | 1.07 ± 0.15 Aa | 0.74 ± 0.09 Aa | 0.07 ± 0.02 ABa | 0.06 ± 0.01 ABa | 0.02 ± 0.01 Aa | 0.06 ± 0.01 Aa | 4.55 ± 0.32 Aab |
|  | UMCH | 41.41 ± 3.99 Aa | 23.90 ± 0.95 ABb | 20.52 ± 3.45 Aab | 4.05 ± 0.89 ABa | 2.05 ± 0.54 Aac | 0.64 ± 0.10 Aab | 1.15 ± 0.34 Aa | 0.11 ± 0.02 ABa | 0.07 ± 0.02 Aa | 0.07 ± 0.02 Aa | 0.13 ± 0.03 Aa | 5.89 ± 0.35 Aa |
| Fine root | PM59 | 20.72 ± 1.60 Ba | 52.70 ± 1.23 Ba | 18.95 ± 1.93 Aa | 2.10 ± 0.93 Ba | 0.03 ± 0.01 Bb | 0.42 ± 0.04 Bb | 0.24 ± 0.05 Ba | 0.38 ± 0.05 Aa | 0.11 ± 0.00 Aa | 0.14 ± 0.12 Aa | 0.04 ± 0.01 Aa | 4.19 ± 0.74 Aa |
|  | UMPM | 22.37 ± 1.28 Ba | 52.45 ± 2.57 Aa | 19.89 ± 2.77 Aa | 0.89 ± 0.24 Ba | 0.08 ± 0.02 Bab | 0.32 ± 0.06 Ab | 0.26 ± 0.10 Ba | 0.48 ± 0.09 Aa | 0.08 ± 0.01 Aa | 0.08 ± 0.01 Aa | 0.09 ± 0.03 Aa | 3.00 ± 0.45 Ba |
|  | CH83 | 26.04 ± 0.97 Ba | 50.28 ± 3.58 Aa | 17.48 ± 3.61 Aa | 2.28 ± 0.90 Ba | 0.15 ± 0.08 Bab | 0.76 ± 0.05 Ba | 0.23 ± 0.06 Ba | 0.29 ± 0.05 Aa | 0.11 ± 0.02 Aa | 0.06 ± 0.03 Aa | 0.09 ± 0.02 Aa | 2.22 ± 0.20 Ba |
|  | UMCH | 21.65 ± 1.12 Ba | 50.32 ± 2.32 Aa | 23.48 ± 2.69 Aa | 0.97 ± 0.30 Ba | 0.24 ± 0.06 Ba | 0.54 ± 0.14 Aab | 0.15 ± 0.09 Ba | 0.34 ± 0.11 Aa | 0.06 ± 0.01 Aa | 0.14 ± 0.08 Aa | 0.08 ± 0.01 Aa | 2.03 ± 0.18 Ba |

**Table S7** The results of the linear mixed-effects model show the effects of plantation type, microhabitat, and their interactions on the relative abundance of bacterial communities at the phylum level.

|  | | Acidobacteria | Proteobacteria | Actinobacteria | Chloroflexi | Firmicutes | Verrucomicrobia | Dependentiae | Armatimonadetes | Gemmatimonadetes | Bacteroidetes | Others | Unclassified |
| --- | --- | --- | --- | --- | --- | --- | --- | --- | --- | --- | --- | --- | --- |
| **Even-aged plantations** | | | | | | | | | | | | | |
| Plantation type | *F* | 1.92 | 0.30 | 0.95 | 1.74 | 4.32 | 3.81 | 1.39 | 4.49 | 2.25 | 0.92 | 1.40 | 0.19 |
|  | *p* | 0.141 | 1.000 | 0.423 | 0.172 | 1.000 | **<0.05** | 0.259 | **<0.01** | 0.095 | 0.440 | 0.255 | 1.000 |
| Microhabitat | *F* | 71.89 | 202.17 | 7.19 | 49.55 | 141.60 | 46.84 | 24.80 | 89.04 | 48.67 | 4.51 | 10.01 | 63.92 |
|  | *p* | **<0.001** | **<0.001** | **<0.01** | **<0.001** | **<0.001** | **<0.01** | **<0.001** | **<0.001** | **<0.001** | **<0.0**5 | **<0.001** | **<0.001** |
| Plantation type × Microhabitat | *F* | 1.18 | 1.48 | 1.87 | 1.16 | 6.18 | 6.02 | 1.49 | 1.38 | 2.30 | 4.53 | 6.24 | 0.79 |
|  | *p* | 0.335 | 0.207 | 0.108 | 0.346 | **<0.001** | **<0.001** | 0.205 | 0.245 | 0.051 | **<0.01** | **<0.001** | 0.580 |
| **Uneven-aged plantations** | | | | | | | | | | | | | |
| Plantation type | *F* | 1.01 | 2.79 | 1.07 | 0.37 | 4.97 | 1.88 | 1.07 | 2.11 | 1.13 | 0.44 | 0.81 | 0.64 |
|  | *p* | 0.393 | 1.000 | 0.368 | 0.773 | **<0.01** | 0.142 | 0.370 | 0.108 | 0.343 | 0.724 | 0.492 | 1.000 |
| Microhabitat | *F* | 78.19 | 373.55 | 2.22 | 54.27 | 122.05 | 11.16 | 31.77 | 55.47 | 8.21 | 4.68 | 1.62 | 30.05 |
|  | *p* | **<0.001** | **<0.001** | 0.118 | **<0.001** | **<0.001** | **<0.001** | **<0.001** | **<0.001** | **<0.001** | **<0.05** | 0.207 | **<0.001** |
| Plantation type × Microhabitat | *F* | 0.96 | 2.26 | 2.16 | 2.22 | 2.61 | 4.19 | 2.45 | 1.68 | 1.81 | 0.81 | 1.47 | 3.86 |
|  | *p* | 0.458 | **<0.05** | 0.059 | 0.053 | **<0.05** | **<0.01** | **<0.05** | 0.142 | 0.112 | 0.567 | 0.203 | **<0.01** |

**Table S8** The relative abundance (%) at the phylum level of fungal communities inhabiting the three microhabitats in different plantations.

| Microhabitat | Plantation type | Basidiomycota | Ascomycota | Mucoromycota | Mortierellomycota | Kickxellomycota | Calcarisporiellomycota | Glomeromycota | Chytridiomycota | Unclassified |
| --- | --- | --- | --- | --- | --- | --- | --- | --- | --- | --- |
| **Even-aged plantations** | | | | | | | | | | |
| Bulk soil | PM83 | 46.82 ± 7.37 Ba | 41.89 ± 6.98 Aa | 3.70 ± 0.94 Aa | 1.56 ± 0.27 Ba | 0.01 ± 0.01 Aa | 0.01 ± 0.00 Aa | 0.00 ± 0.00 Ba | 0.00 ± 0.00 Aa | 6.00 ± 1.46 ABa |
|  | EMPM | 61.78 ± 6.43 Aa | 25.43 ± 5.84 Aa | 3.20 ± 0.81 Aa | 6.07 ± 2.52 Aa | 0.00 ± 0.00 Aa | 0.00 ± 0.00 Ab | 0.02 ± 0.02 Aa | 0.00 ± 0.00 Aa | 3.51 ± 1.49 Aab |
|  | CH83 | 69.21 ± 6.77 Aa | 21.73 ± 4.81 Aa | 2.15 ± 0.64 ABa | 5.70 ± 3.68 Aa | 0.00 ± 0.00 Aa | 0.00 ± 0.00 Aab | 0.00 ± 0.00 Aa | 0.00 ± 0.00 Aa | 1.20 ± 0.14 ABb |
|  | EMCH | 61.78 ± 6.43 Ba | 25.43 ± 5.84 Aa | 3.20 ± 0.81 Aa | 6.07 ± 2.52 Aa | 0.00 ± 0.00 Aa | 0.00 ± 0.00 Ab | 0.02 ± 0.02 Aa | 0.00 ± 0.00 Aa | 3.51 ± 1.49 Aab |
| Rhizosphere soil | PM83 | 82.23 ± 2.36 Aab | 9.38 ± 1.08 Bb | 0.72 ± 0.41 Bb | 4.71 ± 1.14 Aa | 0.01 ± 0.01 Aa | 0.00 ± 0.00 Aa | 0.00 ± 0.00 Ba | 0.00 ± 0.00 Aa | 2.95 ± 0.75 Ba |
|  | EMPM | 70.81 ± 9.53 Ab | 26.18 ± 9.66 Aab | 0.36 ± 0.21 Ab | 1.38 ± 0.26 Aa | 0.00 ± 0.00 Aa | 0.00 ± 0.00 Aa | 0.00 ± 0.00 Aa | 0.00 ± 0.00 Aa | 1.27 ± 0.35 Aab |
|  | CH83 | 45.80 ± 3.17 Bc | 43.15 ± 5.46 Aa | 6.92 ± 2.43 Aa | 3.33 ± 1.97 Aa | 0.01 ± 0.01 Aa | 0.00 ± 0.00 Aa | 0.00 ± 0.00 Aa | 0.00 ± 0.00 Aa | 0.78 ± 0.16Bb |
|  | EMCH | 88.18 ± 0.96 Aa | 7.04 ± 0.19 Bb | 0.08 ± 0.03 Bb | 2.20 ± 0.30 Aa | 0.00 ± 0.00 Aa | 0.00 ± 0.00 Aa | 0.00 ± 0.00 Aa | 0.00 ± 0.00 Aa | 2.50 ± 0.98 Aa |
| Fine root | PM83 | 63.02 ± 4.82 ABa | 26.01 ± 4.21 ABb | 1.11 ± 0.22 Bab | 0.72 ± 0.16 Bbc | 0.00 ± 0.00 Aa | 0.00 ± 0.00 Aa | 0.00 ± 0.00 Aa | 0.00 ± 0.00 Aa | 9.15 ± 1.62 Aa |
|  | EMPM | 40.03 ± 5.86 Ab | 37.82 ± 2.34 Aa | 9.54 ± 7.75 Aa | 1.18 ± 0.27 Aab | 0.00 ± 0.00 Aa | 0.00 ± 0.00 Aa | 0.00 ± 0.00 Aa | 0.00 ± 0.00 Aa | 11.42 ± 3.97 Aa |
|  | CH83 | 75.27 ± 2.93 Aa | 21.66 ± 3.00 Bb | 0.14 ± 0.09 Bb | 0.48 ± 0.15 Bc | 0.06 ± 0.04 Aa | 0.00 ± 0.00 Aa | 0.00 ± 0.00 Aa | 0.00 ± 0.00 Aa | 2.38 ± 0.51 Ab |
|  | EMCH | 76.35 ± 2.68 ABa | 19.44 ± 2.46 ABb | 0.23 ± 0.04 ABab | 1.78 ± 0.21 Aa | 0.00 ± 0.00 Aa | 0.00 ± 0.00 Aa | 0.00 ± 0.00 Aa | 0.00 ± 0.00 Aa | 2.19 ± 0.22 Ab |
| **Uneven-aged plantations** | | | | | | | | | | |
| Bulk soil | PM59 | 57.51 ± 5.35 Aa | 30.34 ± 4.91 Aa | 5.96 ± 2.20 Aa | 2.05 ± 0.25 Aa | 0.01 ± 0.01 Aa | 0.00 ± 0.00 Aa | 0.01 ± 0.01 Aa | 0.01 ± 0.01 Aa | 4.09 ± 1.16 Aa |
|  | UMPM | 50.95 ± 6.12 Ba | 38.29 ± 5.27 Aa | 5.53 ± 1.37 Aa | 3.30 ± 1.16 Aa | 0.00 ± 0.00 Aa | 0.02 ± 0.01 Aa | 0.00 ± 0.00 Aa | 0.00 ± 0.00 Aa | 1.91 ± 0.47 Aab |
|  | CH83 | 69.21 ± 6.77 Aa | 21.73 ± 4.81 Aa | 2.15 ± 0.64 ABa | 5.70 ± 3.68 Aa | 0.00 ± 0.00 Aa | 0.00 ± 0.00 Aa | 0.00 ± 0.00 Aa | 0.00 ± 0.00 Aa | 1.20 ± 0.14 ABb |
|  | UMCH | 50.95 ± 6.12 Ba | 38.29 ± 5.27 Ba | 5.53 ± 1.37 Aa | 3.30 ± 1.16 Aa | 0.00 ± 0.00 Aa | 0.02 ± 0.01 Aa | 0.00 ± 0.00 Aa | 0.00 ± 0.00 Aa | 1.91 ± 0.47 Aab |
| Rhizosphere soil | PM59 | 76.98 ± 4.95 Aa | 14.42 ± 3.91 Bb | 3.64 ± 2.28 ABa | 1.44 ± 0.19 ABa | 0.00 ± 0.00 Ba | 0.00 ± 0.00 Aa | 0.01 ± 0.01 Aa | 0.00 ± 0.00 Aa | 3.51 ± 1.25 Aa |
|  | UMPM | 52.57 ± 4.82 Bab | 38.33 ± 4.09 Aab | 5.72 ± 0.95 Aa | 1.54 ± 0.32 ABa | 0.03 ± 0.03 Aa | 0.01 ± 0.00 ABa | 0.00 ± 0.00 Aa | 0.00 ± 0.00 Aa | 1.81 ± 0.65 Aab |
|  | CH83 | 45.80 ± 3.17 Ba | 43.15 ± 5.46 Ba | 6.92 ± 2.43 Aa | 3.33 ± 1.97 Aa | 0.01 ± 0.01 Aa | 0.00 ± 0.00 Aa | 0.00 ± 0.00 Aa | 0.00 ± 0.00 Aa | 0.78 ± 0.16 Bb |
|  | UMCH | 33.90 ± 7.89 Bb | 56.38 ± 7.24 Aa | 7.67 ± 2.22 Aa | 0.95 ± 0.20 Ba | 0.00 ± 0.00 Aa | 0.00 ± 0.00 Ba | 0.00 ± 0.00 Aa | 0.02 ± 0.02 Aa | 1.08 ± 0.24 Aab |
| Fine root | PM59 | 70.26 ± 3.87 Aa | 20.78 ± 2.62 ABa | 0.27 ± 0.10 Ba | 0.52 ± 0.11 Ba | 0.01 ± 0.00 Aa | 0.00 ± 0.00 Aa | 0.00 ± 0.00 Aa | 0.00 ± 0.00 Aa | 8.16 ± 1.65 Aa |
|  | UMPM | 69.76 ± 5.70 Aa | 27.07 ± 5.31 Aa | 0.04 ± 0.01 Bb | 0.39 ± 0.13 Ba | 0.00 ± 0.00 Aa | 0.00 ± 0.00 Ba | 0.00 ± 0.00 Aa | 0.00 ± 0.00 Aa | 2.74 ± 0.65 Ab |
|  | CH83 | 75.27 ± 2.93 Aa | 21.66 ± 3.00 Aa | 0.14 ± 0.09 Bab | 0.48 ± 0.15 Ba | 0.06 ± 0.04 Aa | 0.00 ± 0.00 Aa | 0.00 ± 0.00 Aa | 0.00 ± 0.00 Aa | 2.38 ± 0.51 Ab |
|  | UMCH | 70.68 ± 3.89 Aa | 24.20 ± 3.27 Ba | 0.04 ± 0.01 Bb | 0.53 ± 0.27 Ba | 0.00 ± 0.00 Aa | 0.00 ± 0.00 Ba | 0.00 ± 0.00 Aa | 0.00 ± 0.00 Aa | 4.55 ± 1.93 Aab |

Note: Values are expressed as mean ± standard error (n = 6). Different lowercase letters within a column indicate significant differences among different plantation types within the same microhabitat. Distinct uppercase letters within a column represent significant differences among different microhabitats within the same plantation. Significance of difference was assessed using the non-parametric Kruskal-Wallis test (*p*  < 0.05). Abbreviations for each plantation type are consistent with those used in Table S2.

**Table S9** The results of the linear mixed-effects model show the effects of plantation type, microhabitat, and their interactions on the relative abundance of fungal communities at the phylum level.

|  | | Basidiomycota | Ascomycota | Mucoromycota | Mortierellomycota | Kickxellomycota | Calcarisporiellomycota | Glomeromycota | Chytridiomycota | Unclassified |
| --- | --- | --- | --- | --- | --- | --- | --- | --- | --- | --- |
| **Even-aged plantations** | | | | | | | | | | |
| Plantation type | *F* | 2.06 | 1.74 | 1.73 | 0.71 | - | - | - | - | 2.23 |
|  | *p* | 0.120 | 0.173 | 0.175 | 0.552 | - | - | - | - | 0.098 |
| Microhabitat | *F* | 3.95 | 1.67 | 16.25 | 10.10 | - | - | - | - | 11.53 |
|  | *p* | **<0.05** | 0.200 | **<0.001** | **<0.001** | **-** | **-** | **-** | **-** | **<0.001** |
| Plantation type × Microhabitat | *F* | 8.38 | 6.09 | 10.47 | 2.10 | - | - | - | - | 1.67 |
|  | *p* | **<0.001** | **<0.001** | **<0.001** | 0.073 | **-** | **-** | - | - | 0.151 |
| **Uneven-aged plantations** | | | | | | | | | | |
| Plantation type | *F* | 1.53 | 1.50 | 0.12 | 0.59 | - | - | - | - | 1.03 |
|  | *p* | 1.000 | 0.223 | 1.000 | 0.623 | - | - | - | - | 0.387 |
| Microhabitat | *F* | 14.02 | 5.46 | 103.12 | 30.46 | - | - | - | - | 12.68 |
|  | *p* | **<0.001** | **<0.01** | **<0.001** | **<0.001** | **-** | **-** | **-** | **-** | **<0.001** |
| Plantation type × Microhabitat | *F* | 4.57 | 4.91 | 3.87 | 0.44 | - | - | - | - | 0.28 |
|  | *p* | **<0.001** | **<0.001** | **<0.01** | 0.852 | **-** | **-** | **-** | - | 0.944 |

Note: -, the relative abundance is zero and the result is not available. The same as below.

**Table S10** Functional composition (%) of bacterial communities inhabiting the three microhabitats in different plantations.

| Microhabitat | Plantation type | Chemoheterotrophy | Aerobic_chemoheterotrophy | Cellulolysis | Iron_respiration | Nitrate_reduction | Intracellular_parasites | Animal_parasites_or_symbionts | Aromatic_compound_degradation | Aromatic_hydrocarbon_degradation | Hydrocarbon_degradation | Others | Unassigned |
| --- | --- | --- | --- | --- | --- | --- | --- | --- | --- | --- | --- | --- | --- |
| **Even-aged plantations** | | | | | | | | | | | | | |
| Bulk soil | PM83 | 8.15 ± 0.52 Aa | 8.12 ± 0.51 Aa | 6.00 ± 0.36 Aa | 0.10 ± 0.02 Ba | 0.22 ± 0.01 Ab | 0.03 ± 0.01 Ba | 0.07 ± 0.02 Aa | 0.01 ± 0.01 Ba | 0.00 ± 0.00 Ba | 0.00 ± 0.00 Ba | 0.64 ± 0.08 ABa | 76.65 ± 1.41 Aa |
|  | EMPM | 7.16 ± 0.90 Aa | 7.14 ± 0.90 Aa | 5.25 ± 0.66 Aa | 0.11 ± 0.02 Aa | 2.37 ± 1.20 Aa | 0.02 ± 0.00 Ba | 0.04 ± 0.01 Aa | 0.02 ± 0.01 Aa | 0.01 ± 0.01 Aa | 0.01 ± 0.01 Aa | 0.55 ± 0.10 Aa | 77.32 ± 1.65 Aa |
|  | CH83 | 7.24 ± 0.84 Aa | 7.23 ± 0.84 Aa | 4.15 ± 0.38 Ba | 0.05 ± 0.01 Ba | 0.28 ± 0.09 Ab | 0.03 ± 0.01 Ba | 0.06 ± 0.02 Aa | 0.00 ± 0.00 Ba | 0.00 ± 0.00 Ba | 0.00 ± 0.00 Ba | 0.72 ± 0.18 Aa | 80.24 ± 1.93 Aa |
|  | EMCH | 7.16 ± 0.90 Ba | 7.14 ± 0.90 Ba | 5.25 ± 0.66 Ba | 0.11 ± 0.02 Ba | 2.37 ± 1.20 Aa | 0.02 ± 0.00 Ba | 0.04 ± 0.01 ABa | 0.02 ± 0.01 Aa | 0.01 ± 0.01 Aa | 0.01 ± 0.01 Aa | 0.55 ± 0.10 Aa | 77.32 ± 1.65 Aa |
| Rhizosphere soil | PM83 | 7.35 ± 1.02 Ba | 7.34 ± 1.01 Ba | 4.74 ± 0.78 Ba | 0.92 ± 0.40 Aa | 0.04 ± 0.01 Ba | 0.11 ± 0.01 Aa | 0.05 ± 0.02 Aa | 0.01 ± 0.01 Ba | 0.01 ± 0.01 ABa | 0.01 ± 0.01 ABa | 0.37 ± 0.13 Ba | 79.05 ± 2.93 Aa |
|  | EMPM | 10.24 ± 1.31 Aa | 10.21 ± 1.32 Aa | 6.80 ± 1.07 Aa | 0.39 ± 0.12 Aa | 0.07 ± 0.03 ABa | 0.15 ± 0.04 ABa | 0.04 ± 0.01 Aa | 0.01 ± 0.01 Aa | 0.01 ± 0.01 Aa | 0.01 ± 0.01 Aa | 0.38 ± 0.08 Aa | 71.69 ± 3.60 Aa |
|  | CH83 | 6.57 ± 0.39 Aa | 6.57 ± 0.39 Aa | 4.86 ± 0.49 ABa | 0.31 ± 0.05 Aa | 0.13 ± 0.07 Aa | 0.07 ± 0.03 ABa | 0.05 ± 0.01 Aa | 0.01 ± 0.01 Aa | 0.01 ± 0.01 Aa | 0.01 ± 0.01 Aa | 0.33 ± 0.13 Aa | 81.10 ± 1.26 Aa |
|  | EMCH | 10.04 ± 1.23 ABa | 10.02 ± 1.22 ABa | 6.91 ± 1.22 ABa | 0.76 ± 0.21 Aa | 0.06 ± 0.02 Ba | 0.15 ± 0.06 Ba | 0.01 ± 0.01 Ba | 0.02 ± 0.01 Aa | 0.02 ± 0.01 Aa | 0.02 ± 0.01 Aa | 0.39 ± 0.16 Aa | 71.60 ± 3.56 ABa |
| Fine root | PM83 | 12.87 ± 0.63 Aa | 12.50 ± 0.65 Aa | 9.31 ± 0.70 Aa | 0.20 ± 0.06 ABa | 0.09 ± 0.05 ABa | 0.06 ± 0.01 ABab | 0.12 ± 0.08 Aa | 0.44 ± 0.05 Aa | 0.43 ± 0.05 Aa | 0.43 ± 0.05 Aa | 0.94 ± 0.37 Aa | 62.60 ± 1.66 Ba |
|  | EMPM | 10.99 ± 1.53 Aa | 9.35 ± 1.02 Aa | 5.96 ± 0.94 Aa | 0.15 ± 0.05 Aa | 0.01 ± 0.00 Ba | 0.30 ± 0.05 Aa | 0.05 ± 0.01 Aa | 0.07 ± 0.01 Aab | 0.07 ± 0.01 Ab | 0.07 ± 0.01 Ab | 0.24 ± 0.03 Aa | 74.29 ± 2.86 Aa |
|  | CH83 | 11.04 ± 1.54 Aa | 10.99 ± 1.53 Aa | 8.51 ± 1.51 Aa | 0.18 ± 0.04 Ba | 0.11 ± 0.05 Aa | 0.14 ± 0.05 Aab | 0.12 ± 0.03 Aa | 0.09 ± 0.03 Aa | 0.07 ± 0.02 Ab | 0.07 ± 0.02 Ab | 0.68 ± 0.16 Aa | 68.01 ± 4.67 Aa |
|  | EMCH | 14.18 ± 2.29 Aa | 14.14 ± 2.31 Aa | 11.76 ± 2.78 Aa | 0.34 ± 0.15 ABa | 0.08 ± 0.02 ABa | 0.19 ± 0.03 Aa | 0.12 ± 0.01 Aa | 0.04 ± 0.01 Ab | 0.04 ± 0.01 Ab | 0.04 ± 0.01 Ab | 0.60 ± 0.24 Aa | 58.49 ± 7.07 Ba |
| **Uneven-aged plantations** | | | | | | | | | | | | | |
| Bulk soil | PM59 | 6.29 ± 0.53 Aa | 6.26 ± 0.53 Aa | 4.07 ± 0.37 Ba | 0.14 ± 0.03 Ba | 0.26 ± 0.04 Aa | 0.03 ± 0.01 Ba | 0.06 ± 0.01 Aa | 0.01 ± 0.01 Ba | 0.01 ± 0.01 Ba | 0.01 ± 0.01 Ba | 0.83 ± 0.10 Aa | 82.04 ± 1.47 Aa |
|  | UMPM | 8.40 ± 1.31 Aa | 8.38 ± 1.30 Aa | 5.83 ± 1.09 Ba | 0.12 ± 0.03 Ba | 0.16 ± 0.05 Aa | 0.03 ± 0.01 Ba | 0.06 ± 0.02 Aa | 0.02 ± 0.01 Aa | 0.02 ± 0.01 Aa | 0.02 ± 0.01 Aa | 0.68 ± 0.06 Aa | 76.28 ± 3.69 Aa |
|  | CH83 | 7.24 ± 0.84 Aa | 7.23 ± 0.84 Aa | 4.15 ± 0.38 Ba | 0.05 ± 0.01 Ba | 0.28 ± 0.09 Aa | 0.03 ± 0.01 Ba | 0.06 ± 0.02 Aa | 0.00 ± 0.00 Ba | 0.00 ± 0.00 Ba | 0.00 ± 0.00 Ba | 0.72 ± 0.18 Aa | 80.24 ± 1.93 Aa |
|  | UMCH | 8.40 ± 1.31 Ba | 8.38 ± 1.30 Ba | 5.83 ± 1.09 Aa | 0.12 ± 0.03 Ba | 0.16 ± 0.05 Aa | 0.03 ± 0.01 Ba | 0.06 ± 0.02 Ba | 0.02 ± 0.01 Aa | 0.02 ± 0.01 Aa | 0.02 ± 0.01 Aa | 0.68 ± 0.06 Aa | 76.28 ± 3.69 Aa |
| Rhizosphere soil | PM59 | 7.66 ± 0.77 Aa | 7.64 ± 0.77 Abc | 5.50 ± 0.83 ABbc | 0.73 ± 0.25 Aa | 0.05 ± 0.01 Aa | 0.11 ± 0.02 ABa | 0.05 ± 0.02 Aa | 0.02 ± 0.01 Aa | 0.02 ± 0.01 Aa | 0.02 ± 0.01 Aa | 0.29 ± 0.07 Aa | 77.91 ± 2.32 Aab |
|  | UMPM | 12.27 ± 1.24 Ab | 12.26 ± 1.24 Aa | 10.79 ± 1.19 Aab | 0.29 ± 0.03 ABa | 0.04 ± 0.02 Aa | 0.10 ± 0.03 ABa | 0.09 ± 0.03 Aa | 0.01 ± 0.00 Ba | 0.00 ± 0.00 Ba | 0.00 ± 0.00 Ba | 0.48 ± 0.09 Aa | 63.66 ± 3.66 Ac |
|  | CH83 | 6.57 ± 0.39 Aa | 6.57 ± 0.39 Ac | 4.86 ± 0.49 ABc | 0.31 ± 0.05 Aa | 0.13 ± 0.07 Aa | 0.07 ± 0.03 ABa | 0.05 ± 0.01 Aa | 0.01 ± 0.01 Aa | 0.01 ± 0.01 Aa | 0.01 ± 0.01 Aa | 0.33 ± 0.13 Aa | 81.10 ± 1.26 Aa |
|  | UMCH | 10.49 ± 1.34 ABab | 10.48 ± 1.34 ABab | 8.76 ± 1.33 Aa | 0.42 ± 0.09 Ba | 0.18 ± 0.04 Aa | 0.06 ± 0.01 ABa | 0.06 ± 0.01 Ba | 0.01 ± 0.01 Ba | 0.00 ± 0.00 Ba | 0.00 ± 0.00 Ba | 0.48 ± 0.10 Aa | 69.06 ± 3.97 ABbc |
| Fine root | PM59 | 12.66 ± 0.88 Aa | 12.49 ± 0.84 Aa | 9.28 ± 0.79 Aa | 0.50 ± 0.16 ABab | 0.04 ± 0.01 Aa | 0.17 ± 0.03 Aa | 0.08 ± 0.01 Aa | 0.19 ± 0.03 Aa | 0.18 ± 0.03 Aa | 0.18 ± 0.03 Aa | 0.44 ± 0.06 Aa | 63.78 ± 2.57 Aa |
|  | UMPM | 12.94 ± 1.38 Aa | 12.76 ± 1.37 Aa | 9.00 ± 1.25 ABa | 0.58 ± 0.15 Aab | 0.05 ± 0.02 Aa | 0.19 ± 0.02 Aa | 0.08 ± 0.02 Aa | 0.19 ± 0.05 Aa | 0.19 ± 0.05 Aa | 0.19 ± 0.05 Aa | 0.46 ± 0.09 Aa | 63.37 ± 3.99 Aa |
|  | CH83 | 11.04 ± 1.54 Aa | 10.99 ± 1.53 Aa | 8.51 ± 1.51 Aa | 0.18 ± 0.04 Bb | 0.11 ± 0.05 Aa | 0.14 ± 0.05 Aa | 0.12 ± 0.03 Aa | 0.09 ± 0.03 Aa | 0.07 ± 0.02 Aa | 0.07 ± 0.02 Aa | 0.68 ± 0.16 Aa | 68.01 ± 4.67 Aa |
|  | UMCH | 14.26 ± 1.17 Aa | 14.20 ± 1.16 Aa | 10.50 ± 0.98 Aa | 0.62 ± 0.17 Aa | 0.16 ± 0.04 Aa | 0.09 ± 0.02 Aa | 0.15 ± 0.02 Aa | 0.11 ± 0.03 Aa | 0.09 ± 0.02 Aa | 0.09 ± 0.02 Aa | 0.73 ± 0.12 Aa | 59.00 ± 3.26 Ba |

Note: Values are expressed as mean ± standard error (n = 6). Different lowercase letters within a column indicate significant differences among different plantation types within the same microhabitat. Distinct uppercase letters within a column represent significant differences among different microhabitats within the same plantation. Significance of difference was determined by non-parametric Kruskal-Wallis test (*p*  < 0.05). Abbreviations for each plantation type are consistent with those used in Table S2.

**Table S11** The results of the linear mixed-effects model show the effects of plantation type, microhabitat, and their interactions on the relative abundance of the functional composition of bacterial communities.

|  | | Chemoheterotrophy | Aerobic_chemoheterotrophy | Cellulolysis | Iron_respiration | Nitrate_reduction | Intracellular_parasites | Animal_parasites_or_symbionts | Aromatic_compound_degradation | Aromatic_hydrocarbon_degradation | Hydrocarbon_degradation | Others | Unassigned |
| --- | --- | --- | --- | --- | --- | --- | --- | --- | --- | --- | --- | --- | --- |
| **Even-aged plantations** | | | | | | | | | | | | | |
| Plantation type | *F* | 1.28 | 1.34 | 1.71 | 2.05 | 0.54 | 2.34 | 0.93 | - | - | - | 0.49 | 1.85 |
|  | *p* | 1.000 | 0.273 | 0.178 | 0.121 | 0.660 | 0.313 | 0.434 | - | - | - | 0.694 | 0.153 |
| Microhabitat | *F* | 15.51 | 14.93 | 12.98 | 40.26 | 28.42 | 64.16 | 8.88 | - | - | - | 3.90 | 18.29 |
|  | *p* | **<0.001** | **<0.001** | **<0.001** | **<0.001** | **<0.001** | **<0.001** | **<0.001** | **-** | **-** | **-** | **<0.05** | **<0.001** |
| Plantation type × Microhabitat | *F* | 1.88 | 1.86 | 1.87 | 0.80 | 2.86 | 2.87 | 1.61 | - | - | - | 0.77 | 2.07 |
|  | *p* | 0.105 | 0.109 | 0.108 | 0.573 | **<0.05** | **<0.05** | 0.167 | - | - | **-** | 0.595 | 0.076 |
| **Uneven-aged plantations** | | | | | | | | | | | | | |
| Plantation type | *F* | 1.49 | 1.48 | 1.49 | 2.26 | 0.97 | 0.84 | 0.50 | - | - | - | 0.62 | 1.53 |
|  | *p* | 0.226 | 0.229 | 0.228 | 0.090 | 0.412 | 0.475 | 0.683 | - | - | - | 0.604 | 0.215 |
| Microhabitat | *F* | 22.19 | 21.51 | 18.77 | 49.93 | 6.98 | 38.74 | 6.24 | - | - | - | 9.90 | 22.66 |
|  | *p* | **<0.001** | **<0.001** | **<0.001** | **<0.001** | **<0.01** | **<0.001** | **<0.01** | **-** | **-** | **-** | **<0.001** | **<0.001** |
| Plantation type × Microhabitat | *F* | 1.17 | 1.19 | 1.81 | 1.30 | 1.69 | 0.34 | 0.97 | - | - | - | 1.04 | 1.30 |
|  | *p* | 0.335 | 0.322 | 0.113 | 0.271 | 0.139 | 0.912 | 0.452 | - | - | - | 0.412 | 0.271 |

**Table S12** Functional composition (%) of fungal communities inhabiting the three microhabitats in different plantations.

| Microhabitat | Plantation type | Symbiotroph | Pathotroph-Saprotroph-Symbiotroph | Saprotroph-Symbiotroph | Saprotroph | Pathotroph-Symbiotroph | Pathotroph | Pathotroph-Saprotroph | Unassigned |
| --- | --- | --- | --- | --- | --- | --- | --- | --- | --- |
| **Even-aged plantations** | | | | | | | | | |
| Bulk soil | PM83 | 28.99 ± 8.46 Aa | 6.40 ± 1.52 Ab | 8.31 ± 1.13 Aa | 5.19 ± 0.91 Aa | 1.22 ± 0.46 Ba | 0.36 ± 0.12 Aa | 0.16 ± 0.09 Aa | 49.37 ± 7.69 ABa |
|  | EMPM | 31.18 ± 6.52 Aa | 21.42 ± 9.70 Aab | 12.87 ± 3.87 Aa | 4.33 ± 1.36 Aa | 1.01 ± 0.34 Ba | 0.21 ± 0.03 Bab | 0.09 ± 0.02 Aa | 28.88 ± 5.06 ABab |
|  | CH83 | 16.72 ± 4.71 Aa | 42.22 ± 8.46 Aa | 16.44 ± 3.89 Aa | 2.26 ± 0.32 Ba | 0.74 ± 0.21 Aa | 0.10 ± 0.02 Ab | 0.11 ± 0.06 Aa | 21.41 ± 5.30 Ab |
|  | EMCH | 31.18 ± 6.52 Aa | 21.42 ± 9.70 Aab | 12.87 ± 3.87 Aa | 4.33 ± 1.36 Aa | 1.01 ± 0.34 A | 0.21 ± 0.03 Aa | 0.09 ± 0.02 Aa | 28.88 ± 5.06 Aab |
| Rhizosphere soil | PM83 | 31.91 ± 15.83 Aa | 35.26 ± 10.13 Aa | 0.21 ± 0.09 Aa | 2.85 ± 0.56 ABab | 12.30 ± 2.99 Aab | 0.16 ± 0.04 Aa | 0.02 ± 0.00 Aa | 17.19 ± 4.98 Bab |
|  | EMPM | 54.92 ± 20.99 Aa | 20.32 ± 14.83 Aa | 0.18 ± 0.03 Aa | 1.73 ± 0.80 Aab | 15.53 ± 5.74 Aa | 0.25 ± 0.11 ABa | 0.02 ± 0.01 Ba | 7.14 ± 1.05 Bb |
|  | CH83 | 30.88 ± 5.28 Aa | 29.02 ± 3.32 Aa | 1.39 ± 0.66 Aa | 7.96 ± 2.60 Aa | 9.09 ± 3.53 Ab | 0.11 ± 0.04 Aa | 0.01 ± 0.01 Aa | 21.54 ± 1.99 Aa |
|  | EMCH | 43.96 ± 14.42 Aa | 16.13 ± 6.98 Aa | 0.74 ± 0.13 Aa | 1.26 ± 0.12 Ab | 26.29 ± 8.10 Aa | 0.25 ± 0.05 Aa | 0.01 ± 0.00 Ba | 11.35 ± 0.59 Bab |
| Fine root | PM83 | 7.41 ± 2.88 Ab | 32.57 ± 7.95 Aa | 7.56 ± 1.28 Aa | 1.44 ± 0.36 Ba | 0.18 ± 0.04 Ba | 0.19 ± 0.03 Aa | 0.05 ± 0.03 Aa | 50.60 ± 6.93 Aa |
|  | EMPM | 17.64 ± 3.24 Aab | 6.95 ± 1.89 Aa | 22.09 ± 7.53 Aa | 4.09 ± 0.46 Aa | 0.20 ± 0.03 Ba | 0.88 ± 0.35 Aa | 0.04 ± 0.01 ABa | 48.12 ± 4.54 Aa |
|  | CH83 | 20.40 ± 6.47 Aab | 30.25 ± 6.89 Aa | 22.58 ± 4.16 Aa | 3.32 ± 0.86 ABa | 0.30 ± 0.16 Aa | 0.22 ± 0.07 Aa | 0.06 ± 0.03 Aa | 22.87 ± 6.19 Aa |
|  | EMCH | 53.32 ± 13.28 Aa | 10.37 ± 3.74 Aa | 18.12 ± 7.75 Aa | 2.88 ± 0.45 Aa | 0.38 ± 0.07 Aa | 0.47 ± 0.23 Aa | 0.01 ± 0.01 Ba | 14.45 ± 1.84 ABa |
| **Uneven-aged plantations** | | | | | | | | | |
| Bulk soil | PM59 | 14.85 ± 4.27 Aa | 8.18 ± 1.33 Ab | 31.94 ± 7.91 Aa | 6.30 ± 2.15 Aa | 1.33 ± 0.31 Aa | 0.28 ± 0.06 Aab | 0.17 ± 0.04 Aa | 36.94 ± 3.81 ABa |
|  | UMPM | 13.16 ± 2.35 Aa | 20.14 ± 6.76 Aab | 17.50 ± 2.40 Aa | 6.41 ± 1.35 Aa | 1.94 ± 0.39 Aa | 0.45 ± 0.11 Aa | 0.20 ± 0.08 Aa | 40.21 ± 5.94 Aa |
|  | CH83 | 16.72 ± 4.71 Aa | 42.22 ± 8.46 Aa | 16.44 ± 3.89 ABa | 2.26 ± 0.32 Bab | 0.74 ± 0.21 Aa | 0.10 ± 0.02 Ab | 0.11 ± 0.06 Aa | 21.41 ± 5.30 Aa |
|  | UMCH | 13.16 ± 2.35 Aa | 20.14 ± 6.76 Aab | 17.50 ± 2.40 Aa | 6.41 ± 1.35 Aa | 1.94 ± 0.39 Aa | 0.45 ± 0.11 Aa | 0.20 ± 0.08 ABa | 40.21 ± 5.94 Aa |
| Rhizosphere soil | PM59 | 20.48 ± 7.09 Aa | 9.16 ± 1.24 Abc | 41.74 ± 8.47 Aa | 4.71 ± 1.85 Aa | 0.80 ± 0.49 ABa | 0.24 ± 0.05 Ab | 0.04 ± 0.01 Bab | 22.82 ± 5.44 Ba |
|  | UMPM | 15.21 ± 4.82 Aa | 16.27 ± 3.43 Ab | 22.39 ± 5.54 Aab | 7.50 ± 0.66 Aa | 0.52 ± 0.10 ABa | 0.45 ± 0.18 Aa | 0.02 ± 0.01 Bb | 37.64 ± 4.64 Aa |
|  | CH83 | 30.88 ± 5.28 Aa | 29.02 ± 3.32 Aa | 9.09 ± 3.53 Bb | 7.96 ± 2.60 Aa | 1.39 ± 0.66 Aa | 0.11 ± 0.04 Ab | 0.01 ± 0.01 Ab | 21.54 ± 1.99 Aa |
|  | UMCH | 32.07 ± 7.40 Aa | 4.92 ± 0.76 Bc | 16.88 ± 5.83 Aab | 6.30 ± 1.01 Aa | 0.66 ± 0.17 ABa | 0.44 ± 0.13 Bab | 0.05 ± 0.03 Ba | 38.68 ± 8.36 Aa |
| Fine root | PM59 | 10.13 ± 2.27 Aab | 20.96 ± 5.32 Aa | 20.57 ± 3.64 Aa | 3.16 ± 1.67 Aa | 0.15 ± 0.03 Ba | 0.44 ± 0.11 Aab | 0.02 ± 0.01 Bb | 44.58 ± 5.68 Aa |
|  | UMPM | 8.49 ± 2.72 Ab | 20.00 ± 4.93 Aa | 12.59 ± 2.47 Aa | 2.61 ± 1.32 Aa | 0.06 ± 0.02 Ba | 1.07 ± 0.57 Aab | 0.06 ± 0.02 ABa | 55.11 ± 6.77 Aa |
|  | CH83 | 20.40 ± 6.47 Aab | 30.25 ± 6.89 Aa | 22.58 ± 4.16 Aa | 3.32 ± 0.86 ABa | 0.30 ± 0.16 Aa | 0.22 ± 0.07 Ab | 0.06 ± 0.03 Aa | 22.87 ± 6.19 Aab |
|  | UMCH | 30.49 ± 10.05 Aa | 20.95 ± 8.29 Aa | 22.18 ± 8.03 Aa | 1.62 ± 0.52 Ba | 0.10 ± 0.04 Ba | 2.67 ± 1.18 Aa | 1.12 ± 0.43 Aa | 20.88 ± 3.38 Ab |

Note: Values are expressed as mean ± standard error (n = 6). Different lowercase letters within a column indicate significant differences among different plantation types within the same microhabitat. Distinct uppercase letters within a column represent significant differences among different microhabitats within the same plantation. Significance of difference was determined by non-parametric Kruskal-Wallis test (*p*  < 0.05). Abbreviations for each plantation type are consistent with those used in Table S2.

**Table S13** The results of the linear mixed-effects model show the effects of plantation type, microhabitat, and their interactions on the relative abundance of the functional composition of fungal communities.

|  | | Symbiotroph | Pathotroph-Saprotroph-Symbiotroph | Saprotroph-Symbiotroph | Saprotroph | Pathotroph-Symbiotroph | Pathotroph | Pathotroph-Saprotroph | Unassigned |
| --- | --- | --- | --- | --- | --- | --- | --- | --- | --- |
| **Even-aged plantations** | | | | | | | | | |
| Plantation type | *F* | 1.27 | 0.76 | 0.79 | 0.83 | 1.25 | 1.19 | 0.75 | 2.03 |
|  | *p* | 0.296 | 0.521 | 0.508 | 0.484 | 0.302 | 0.325 | 0.525 | 0.124 |
| Microhabitat | *F* | 3.15 | 0.61 | 1.31 | 2.11 | 10.24 | 4.13 | 9.11 | 20.61 |
|  | *p* | 0.052 | 0.547 | 0.279 | 0.133 | **<0.001** | **<0.05** | **<0.001** | **<0.001** |
| Plantation type × Microhabitat | *F* | 1.46 | 1.96 | 2.14 | 5.50 | 2.46 | 1.60 | 1.05 | 6.01 |
|  | *p* | 0.213 | 0.093 | 0.068 | **<0.001** | **<0.05** | 0.170 | 0.407 | **<0.001** |
| **Uneven-aged plantations** | | | | | | | | | |
| Plantation type | *F* | 1.01 | 0.92 | 1.04 | 0.45 | 0.25 | 0.94 | 1.71 | 1.38 |
|  | *p* | 0.396 | 0.438 | 0.383 | 0.722 | 0.863 | 0.426 | 0.175 | 0.258 |
| Microhabitat | *F* | 3.24 | 3.19 | 0.51 | 15.72 | 50.80 | 6.46 | 15.05 | 0.90 |
|  | *p* | **<0.05** | **<0.05** | 0.604 | **<0.001** | **<0.001** | **<0.01** | **<0.001** | 0.412 |
| Plantation type × Microhabitat | *F* | 0.94 | 1.94 | 2.15 | 2.65 | 2.75 | 0.79 | 4.65 | 3.44 |
|  | *p* | 0.476 | 0.088 | 0.061 | **<0.05** | **<0.05** | 0.580 | **<0.001** | **<0.01** |

**Table S14** Topological properties of bacterial and fungal co-occurrence networks in each microhabitat.

| Microhabitat | Number of nodes | Number of edges | Positive edges (%) | Negative edges (%) | Average degree (avgK) | Average clustering coefficient  (avgCC) | Average  path distance  (GD) | Modularity | Transitivity (Trans) | Connectance (Con) | Efficiency | Hub nodes | | |
| --- | --- | --- | --- | --- | --- | --- | --- | --- | --- | --- | --- | --- | --- | --- |
|  |  |  |  |  |  |  |  |  |  |  |  | Connector hubs | Module hubs | Network hubs |
| **Bacteria Even-aged plantations** | | | | | | | | | | | | | | |
| Bulk soil | 383 | 2139 | 74.24 | 25.76 | 11.170 | 0.329 | 3.401 | 0.443 | 0.361 | 0.949 | 0.972 | 34 | 8 | 1 |
| Rhizosphere soil | 504 | 6546 | 56.72 | 43.28 | 25.976 | 0.401 | 2.843 | 0.327 | 0.428 | 0.976 | 0.949 | 19 | 9 | 0 |
| Fine root | 396 | 2990 | 57.63 | 42.37 | 15.101 | 0.315 | 3.027 | 0.358 | 0.353 | 0.980 | 0.963 | 54 | 2 | 0 |
| **Bacteria Uneven-aged plantations** | | | | | | | | | | | | | | |
| Bulk soil | 410 | 2906 | 70.96 | 29.04 | 14.176 | 0.385 | 3.246 | 0.474 | 0.382 | 0.990 | 0.967 | 20 | 11 | 0 |
| Rhizosphere soil | 389 | 1688 | 64.51 | 35.49 | 8.679 | 0.288 | 3.741 | 0.459 | 0.353 | 0.890 | 0.978 | 20 | 9 | 0 |
| Fine root | 300 | 1094 | 63.99 | 36.01 | 7.293 | 0.267 | 3.696 | 7.293 | 0.311 | 0.947 | 0.978 | 20 | 6 | 0 |
| **Fungi Even-aged plantations** | | | | | | | | | | | | | | |
| Bulk soil | 103 | 491 | 51.53 | 48.47 | 9.534 | 0.409 | 3.108 | 0.240 | 0.561 | 0.851 | 0.900 | 0 | 2 | 0 |
| Rhizosphere soil | 135 | 1246 | 52.01 | 47.99 | 18.459 | 0.525 | 2.506 | 0.257 | 0.537 | 0.971 | 0.865 | 2 | 0 | 0 |
| Fine root | 119 | 532 | 56.39 | 43.61 | 8.941 | 0.365 | 3.191 | 0.338 | 0.481 | 0.967 | 0.930 | 9 | 0 | 0 |
| **Fungi Uneven-aged plantations** | | | | | | | | | | | | | | |
| Bulk soil | 116 | 227 | 59.03 | 40.97 | 3.914 | 0.221 | 4.073 | 0.632 | 0.341 | 0.775 | 0.966 | 0 | 0 | 0 |
| Rhizosphere soil | 113 | 302 | 59.93 | 40.07 | 5.345 | 0.379 | 3.476 | 0.547 | 0.437 | 0.784 | 0.949 | 1 | 1 | 0 |
| Fine root | 74 | 117 | 60.68 | 39.32 | 3.162 | 0.190 | 4.071 | 0.635 | 0.339 | 0.702 | 0.955 | 2 | 0 | 0 |
